# Deep Generative Model of Macrophage Immune Response for Hepato-intestinal Tumor Therapy Optimization

**DOI:** 10.34133/cbsystems.0559

**Published:** 2026-06-16

**Authors:** Lei Zhou, Yingjie Tan, Weigang Lv, Kening Lin, Feifeng Li, Wen Ouyang, Di Zhang

**Affiliations:** ^1^ Precision Diagnosis Center, The Third Xiangya Hospital, Central South University, Changsha 410013, P.R. China.; ^2^ Department of Anesthesiology, The Third Xiangya Hospital, Central South University, Changsha 410013, P.R. China.; ^3^ Prenatal Diagnosis Center, Department of Obstetrics and Gynecology, The Third Xiangya Hospital, Central South University, Changsha 410013, P.R. China.; ^4^ Department of Clinical Laboratory, The Third Xiangya Hospital, Central South University, Changsha 410013, P.R. China.; ^5^ Department of Pathology, The Third Xiangya Hospital, Central South University, Changsha 410013, P.R. China.; ^6^ Hunan Province Key Laboratory of Brain Homeostasis, The Third Xiangya Hospital, Central South University, Changsha 410013, P.R. China.

## Abstract

Digestive tract cancers, including hepatobiliary and gastrointestinal malignancies, remain a major oncological burden globally. Immunotherapy efficacy rates are low, with only 15% to 30% of patients experiencing responses following treatment. Tumor-associated macrophages, which change phenotype between a pro-inflammatory and an immunosuppressive state, play a key role in determining the response to therapy, and current static biomarkers are inadequate for capturing the spatial–temporal changes associated with the immune response. We developed a bioinspired digital twin platform integrating variational representation learning with causal sequence modeling. The platform incorporates heterogeneous biological data (1.2 million single-cell transcriptomes, spatial immunophenotyping, and clinical trajectories) from 2,847 individuals across 5 digestive cancer types. Graph-based attention mechanisms encode intercellular interactions, while transformer-based temporal modules simulate immunological state transitions. A model-predictive optimization layer identifies patient-specific interventions maximizing repolarization potential. The biomimetic model predicted the outcome of therapy response better than conventional biomarker models did (area under the receiver operating characteristic curve: 0.847 compared to 0.692 with a statistically significant difference at *P* below 0.001). In an exploratory, nonrandomized analysis of discordant cases (*n* = 156) where model and physician recommendations differed, model-guided treatment was associated with higher response rates (47.4% versus 28.2%) and longer median progression-free survival (9.8 months versus 6.0 months; *P* = 0.003); however, selection bias cannot be excluded. This study provides preliminary evidence for the feasibility of a computational framework for immunotherapy optimization; prospective randomized trials are required to establish clinical utility.

## Introduction

Identifying and targeting major gastrointestinal (GI) malignancies, including hepatobiliary malignancies and malignant intestinal cancers, will be necessary to alleviate a major burden to global health with more than 4.8 million new diagnoses and over 3.4 million deaths annually [1]. The introduction of immune checkpoint inhibitors (ICIs) to treat these malignancies has changed the treatment landscape [2–5], but only 15% to 30% of patients experience durable clinical benefit, indicating that these therapies are not particularly effective for most patients [6]. This low efficacy may be due to the highly complex and heterogeneous nature of the tumor microenvironment (TME), where tumor-associated macrophages (TAMs) play a critical role in modulating the immune response and the therapeutic outcome [7].

TAMs are characterized by their extraordinary plasticity, existing along a spectrum of phenotypes ranging from classically activated M1 macrophages (antitumor) to alternatively activated M2 macrophages (profiber growth and immune escape) [8]. In this regard, a dynamic balance between M1-dominant and M2-dominant TMEs will have a critical impact on how patients respond to ICI therapy, with M1 TMEs associated with improved outcomes and M2 TMEs with therapeutic resistance [9]. To address this knowledge gap, it is critical for precision oncology to develop a better understanding of how immunological dynamics evolve temporally, both between patients and over time for individual patients, as most current clinical decisions are made solely based on static biomarkers [10,11] that do not reflect the dynamic evolution of the immune landscape [12].

Newly developed single-cell technologies and multiplex imaging have provided unprecedented ability to characterize TME heterogeneity at the single-cell level [13–17] and generate ultrahigh-dimensional datasets that contain a wealth of information about cellular phenotypes and spatial organization, as well as the networks of communication between cells. Thus, a critical barrier preventing the effective translation of these highly complex datasets into clinically useful predictive models is the lack of computational frameworks that build and utilize generalizable representations of immune dynamics from this information while accounting for the considerable variability observed between patients and cancer types. Although traditional machine learning has shown moderate success in the prediction of response to ICI therapy, the reliance on handcrafted feature engineering has limited the effectiveness of this technology in developing predictive models because it is not possible to model temporal trajectories that dictate treatment outcomes.

Latent dynamics models, originally conceptualized in the reinforcement learning (RL) literature to build predictive environmental representations, have inspired offline generative approaches for modeling complex sequential decision problems [18–21]. Learning how to simulate future states that occur when actions and observations are experienced will allow for planning and optimizing complex partially observable environments. Recently developed deep generative modeling techniques have shown great potential for understanding the relationships in biological systems, including proteins and cell fate trajectories. The concepts of world modeling can be applied to optimize the application of immunotherapies and use generative models as predictive tools while maintaining interpretability through latent representations.

This article presents GI-ImmunoWorld, a deep generative latent dynamics model—inspired by but distinct from online RL world models—that learns representations of macrophage immune dynamics and simulates treatment responses in patients with hepatobiliary–intestinal tumors. The GI-ImmunoWorld framework will help to address 3 core problems of computational immunotherapy. The first is our use of a hierarchical variational autoencoder (VAE) to obtain and represent the latent variable states of TAMs along with cytokine and TME gradient profiles using multimodal patient datasets. Second, we have integrated a transformer-based temporal dynamics module (TDM) to facilitate the modeling of the evolution of immune landscape changes from one therapeutic intervention to another, thereby predicting the potential trajectory of treatment responses. Third, we have added an optimization layer that uses the world model to identify customized optimum therapeutic strategies that maximize the predicted probability of response, taking into account individual patient constraints.

**Fig. 1.**
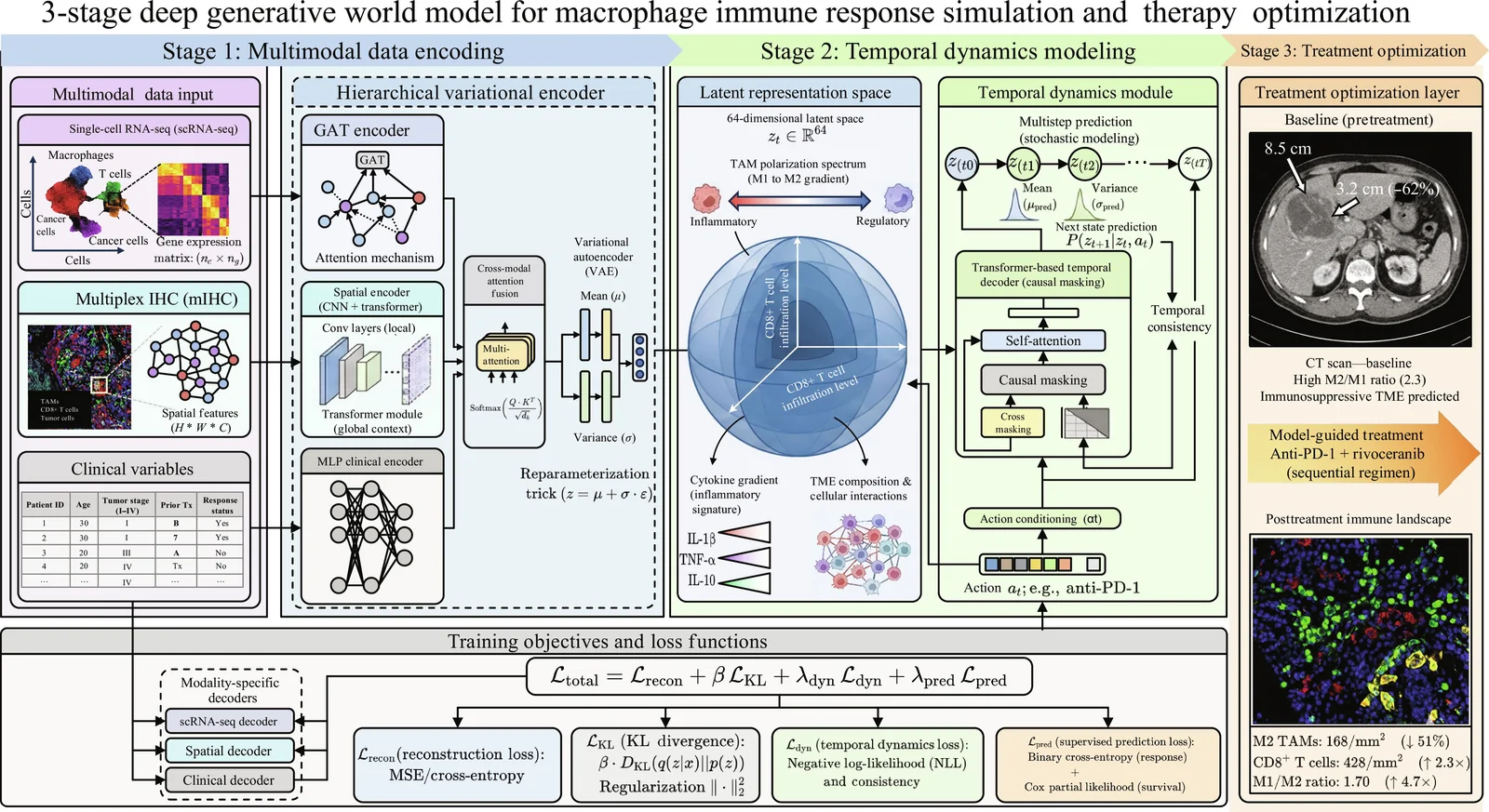
The GI-ImmunoWorld architecture consists of 3 integrated components: (a) the use of a hierarchical, variational encoder to encode multimodal (multiple types of) data through modality-specific encoders (graph attention network [GAT], convolutional neural network [CNN]–transformer, and multilayer perceptron [MLP]) into a single 64-dimensional (64D) latent space using cross-modality attention, (b) the use of a causal transformer decoder to model temporal dynamics and predict the future evolution of the immune system given treatment actions, and (c) a treatment optimization layer using model-predictive control (MPC) and the cross-entropy method (CEM) to identify patient-specific treatment strategies. The latent space has biologically interpretable axes (e.g., the tumor-associated macrophage [TAM] M1/M2 polarization spectrum, CD8+ T cell infiltration levels, and the cytokine gradient signature) that can be used to understand the immune response. An example case demonstrates that the model-guided treatment strategy of anti-programmed cell death protein 1 (anti-PD-1) and rivoceranib resulted in favorable changes in the microenvironment of the immune system.

The design of GI-ImmunoWorld is illustrated in Fig. 1 and encompasses 3 main phases of the framework, which include the encoding of multimodal data, modeling of the temporal dynamics of the immune system, and treatment optimization.

Training and validation of GI-ImmunoWorld was completed using multi-institutional datasets of 2,847 patients with 5 different types of major GI tumors (hepatocellular carcinoma, cholangiocarcinoma, colorectal cancer [CRC], gastric cancer, and pancreatic ductal adenocarcinoma [PDAC]). The training data included 1,200,000 single-cell RNA sequencing (scRNA-seq) profiles, multiplex immunohistochemistry (mIHC) images providing the quantification of the spatial immune architecture of each tumor type, longitudinal clinical outcome data including response assessments and survival data, and a comprehensive account of patient treatment history. The multimodal integration of these datasets enabled the model to learn a unified representation that links molecular signature immune signatures to macroscopic clinical phenotype.

Our experimental studies show that GI-ImmunoWorld outperforms all prior methods across a variety of predictive tasks. For recommendations on immunotherapy response, the model had an area under the receiver operating characteristic curve (AUROC) score of 0.847, with a relative improvement of 22.4% compared to traditional methods that use biomarkers to make recommendations (AUROC = 0.692), and the differences were highly statistically significant (P<0.001). In an exploratory, nonrandomized retrospective simulation using temporally held-out data where model and clinician recommendations diverged (*n* = 156), patients whose clinicians chose to follow the model’s recommendations showed higher objective response rates (ORRs) (47.4% vs 28.2%, P=0.003) and longer median progression-free survival (PFS; 9.8 months vs 6.0 months); these associations are hypothesis generating and subject to potential selection bias. However, caution is warranted regarding any causal inferences that could be drawn from these results, as there is a potential for selection bias to have influenced the associations observed in this exploratory deployment study. In addition, patients who had received model-guided treatment timing were found to have markedly increased CD8+ T cell infiltration (2.3-fold increase) and decreased levels of immunosuppressive TAM signatures, which is consistent with the model representation of this immune response.

The key contributions of this work are listed below. Few prior studies (to our knowledge) have framed the problem of macrophage-centric immunotherapy optimization in GI cancers as a problem of deep generative world modeling; we show that generative modeling of immune dynamics allows for superior predictions about treatment than do discriminative modeling approaches. We also provide a theoretical framework that takes standard variational inference and error propagation bounds as applied to spatiotemporal representations of immunology, and we provide detailed curation and definition of a multimodal extensive evaluation protocol, including partially shared omics databases and software for benchmarking purposes under data-use agreements. Lastly, we provide preliminary evidence from an exploratory prospective deployment study that model-guided treatment recommendations may be correlated with improved patient outcomes, demonstrating the potential for the clinical utility of computational immunological modeling and justifying further testing in the context of randomized controlled trials.

## Materials and Methods

### Patient cohorts and data acquisition

GI-ImmunoWorld was created and validated through the analysis of curated retrospective, multimodal data (clinical, imaging, genomic, etc.) assembled from publicly available repositories for a total of 2,847 individuals diagnosed with hepatobiliary–intestinal malignancies, originally treated from January 2018 to December 2023 across multiple academic medical centers. The date for data locking for the current analysis is 2025 March 31. The study sample consists of a total of 5 cancer types, namely, hepatocellular carcinoma (*n* = 684), cholangiocarcinoma (*n* = 412), CRC (*n* = 892), gastric cancer (*n* = 523), and PDAC (*n* = 336). All patients had received at least one dose of immunotherapy (to include anti-programmed cell death protein 1/programmed death-ligand 1 [anti-PD-1/PD-L1] as monotherapy or in combination with anti-cytotoxic T-lymphocyte-associated protein 4 [anti-CTLA-4], targeted therapies, or other combination regimens). All data were derived exclusively from publicly available repositories (Gene Expression Omnibus [GEO], The Cancer Genome Atlas [TCGA], The Cancer Imaging Archive [TCIA], and cBioPortal) where the original contributing studies had obtained appropriate institutional ethical approvals and informed consent. As this study constitutes a secondary analysis of de-identified public data, no additional institutional review board approval was required per standard institutional policies for retrospective analyses of publicly available datasets.

The multimodal data were assembled from publicly available repositories and previously published datasets; no proprietary or unpublished clinical data were used in this study. The integrated dataset encompasses diverse patient populations from studies conducted across multiple geographic regions, including East Asia, North America, and Europe. This variability in data collection process enhanced the robustness of the study results by eliminating platform-specific artifacts. The external validation cohort was obtained from 3 independent geographic and institutional locations and did not participate in the model development using a different laboratory workflow.

Survival analyses were conducted only with patients who started immunotherapy before 2024 March 31 to ensure that all included patients had a follow-up period of at least 12 months. Patients who began therapy after 2024 March 31 were included in the response prediction analyses but were excluded from PFS and overall survival (OS) analyses in order to preserve the integrity of follow-up data.

The 10x Genomics Chromium platform was used to produce scRNA-seq data for tumor tissue samples. After the quality control steps were completed, filtering based on the percentage of mitochondrial genes (<20%) and the number of genes detected (>500) and identification of doublets using the Scrublet [22] program, the final dataset consisted of 1,247,832 high-quality cells from 1,423 patients (only one tumor sample per patient; matched adjacent normal tissue was collected but analyzed separately and is therefore not included in the total cell count). The annotation of cell types was performed by the following methods: hierarchical clustering using known marker gene signatures and manual curation by expert pathologists. The different populations of TAMs were identified according to the canonical markers CD68, CD163, CD206 (M2 associated), CD80, CD86, and inducible nitric oxide synthase (iNOS) (M1 associated).

mIHC was performed on formalin-fixed paraffin-embedded tissue sections using validated antibody panels to characterize the immune cells within the tissue samples. The primary mIHC panel contained markers for TAMs (CD68, CD163, CD206, and iNOS) and T lymphocytes (CD3, CD8, CD4, forkhead box P3 [FOXP3], and PD-1), along with tumor cells (pan-cytokeratin). For each patient, whole-slide images were taken at a 20× magnification and the HALO platform was used to segment and document the regions of interest for automation analysis of cells based on different phenotypes. The spatial metrics created from HALO analysis captured the number of immune and tumor cells present per square millimeter (density) for each of the following regions: core tumor, invasive margin, and stroma. Proximity scores calculated by the HALO platform provided measures of immune and tumor cell interactions within the various regions of the tumor.

Longitudinal clinical data were obtained from the electronic health records of the patients and included demographics, staging of the tumors, prior treatment histories, treatment regimens, and RECIST v1.1 response assessments. PFS was defined as the time from the start of treatment until the patient had progressed or died from any cause. OS was calculated from the start of treatment to the date of death from any cause or last date in the patient follow-up record. For the purpose of modeling the temporal dynamics of the tumors within the study population, each patient underwent serial clinical and imaging assessments (at least 3) with at least one of the serial assessments having scRNA-seq data available (n=1,423 patients) at each time point. Histopathologic and imaging features were available for all patients at each of the time points in the study.

#### Data completeness and modality coverage

Among 2,847 patients included in this analysis, 1,423 (50.0%) had scRNA-seq data, 2,156 patients (75.7%) had mIHC spatial profiles, and every patient had clinical variables; 1,186 patients (41.7%) had all 3 modalities available for the analysis of the primary modal. The model was developed with modality-specific encoders and missing-modality masks during training and allowed for deployment based upon available data for patients that were missing one of the modalities. Stratified performance analyses across different modality combinations (all 3 modalities, 2 modalities, and single modality) are reported in Table S1 to characterize model robustness under clinically realistic data availability scenarios. The 89 patients who had a serial biopsy were excluded from the development of the model in order to allow for unbiased evaluation of the trajectory predictions of patients.

For the baseline gene expression signature (Tumor Inflammation Signature, TIDE, and ImmuneCells.Sig), we created pseudobulk expression profiles from aggregating scRNA-seq counts from all cells from each patient with available scRNA-seq data in order to compare performance among these methods and other modalities according to the number of patients with available scRNA-seq data in the test data.

#### Prospective deployment cohort

To assess the potential clinical utility of the GI-ImmunoWorld model, we conducted an exploratory retrospective simulation using temporally held-out data. This analysis is strictly hypothesis generating; the nonrandomized design precludes causal inference. Patients from publicly available datasets with treatment dates after December 2023 (*n* = 312) were used to simulate a prospective validation scenario, and all model parameters were held constant.

The main analysis of treatment optimization focused on the “discordant cohort”, which consisted of patients whose first therapy selection diverged from the model’s highest therapeutic suggestion (*n* = 156, 50% of 312). The remaining 156 patients whose model and physician recommendations matched were excluded from all comparative evaluations since these do not provide insights regarding whether following guidance increases or decreases the likelihood of benefit. Among the discordant cohort, 78 patients were assigned by the treating physician (after a review of the model output) to receive a treatment regimen as guided by the model and referred to here as the “model-guided group”, while the other 78 patients were treated with their original standard-of-care regimen and referred to here as the “standard group”. This model-guided versus standard allocation was made observationally and not randomly; indeed, in the latter case, we acknowledge that physicians may choose to adhere to the model recommendation not necessarily because they believed that another therapeutic selection would provide inferior outcomes but also due to specific aspects of the patient profile that were provided through review of the model output.

Of those patients receiving a model-guided therapy allocation, a subset (*n* = 42) had available paired pre- and posttreatment biopsy data in public repositories, enabling mechanistic assessment (this subset is biased toward patients with a more favorable performance status and accessible lesions). All evaluations of prospective data are exploratory and generate/risk hypothesis, as the nonrandomized nature of study design and the potential for bias of selection on part of treating physicians limit the ability to attribute cause.

### GI-ImmunoWorld architecture

**Fig. 2.**
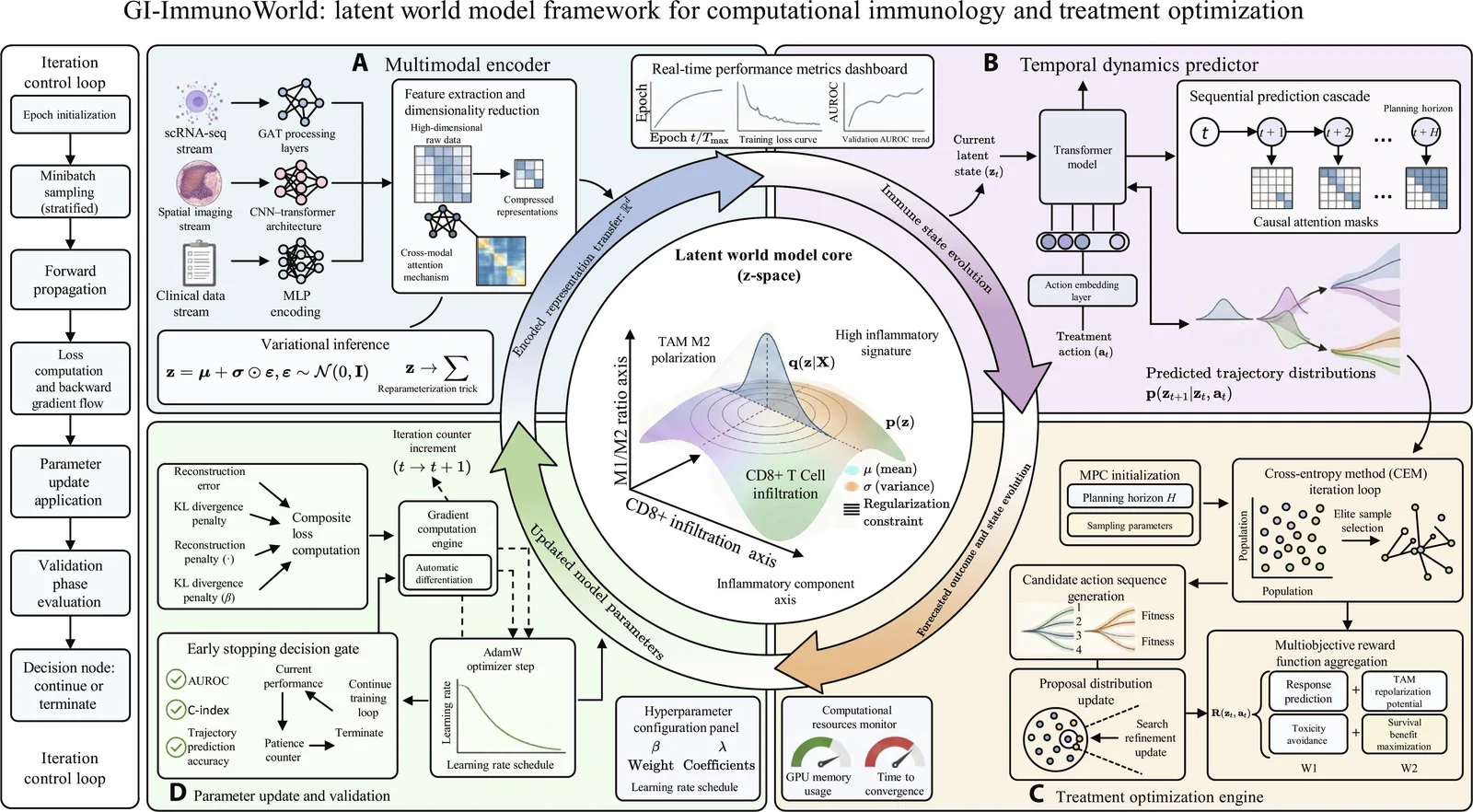
The framework for GI-ImmunoWorld’s latent world model describes computational immunology and treatment optimization. It has a 4-component model architecture. (A) Multimodal encoder: processes multiple data streams from single-cell RNA sequencing (scRNA-seq), using graph attention networks (GATs) to represent the scRNA-seq data and convolutional neural network (CNN)–transformer architectures to encode spatially imaged data as well as using multilayer perceptron (MLP) encoding to represent clinical data. This component fuses the different modalities of data using cross-modal attention and performs variational inference using the reparameterization trick: (z=μ+σ⊙ε), where ε∼N(0,I). (B) Temporal dynamics predictor: the sequential prediction cascade, which uses a transformer-based temporal decoder with causal attention masks, providing the prediction of the distribution of the next time step’s trajectory ($p\left(z_{t+1}|z_{t},\;a_{t}\;\right)$) conditioned on the action taken. (C) Treatment optimizer: model-predictive control (MPC) with an iterative loop of the cross-entropy method (CEM), to provide a candidate sequence of actions, to select elite samples, and to combine the multiobjective reward function that balances the prediction of the response, repolarization potential of a target antigen, avoidance of toxicity, and the survival benefits of the treatment. (D) Parameter update and validation: computes the composite loss consisting of reconstruction loss, Kullback–Leibler (KL) divergence, dynamics loss, and supervised prediction loss. An early stopping decision is made using area under the receiver operating characteristic curve (AUROC), concordance index (C-index), and accuracy of trajectory prediction decisions.

The GI-ImmunoWorld framework consists of 3 mutually connected and interdependent components. A hierarchical encoder is used to fuse multimodal data and to learn latent representations, while a transformer model is used to estimate the immune trajectory evolving as time progresses. Additionally, there is an additional layer that optimizes therapy and provides a personalized recommendation for treatment; see Fig. 2.

These models work together to form a generative world model of the dynamics of the macrophage immune response in the TME and to approximate the temporal evolution of TAM polarization states and interactions between tumors and the immune system.

#### Hierarchical variational encoder

A variational encoder module processes heterogeneous multimodal inputs to form the representations of patient immune states in a latent space. These patient states consist of scRNA-seq data denoted as $X^{\left(s\right)}$, which has the dimensions ${\mathbb{R}}^{n_{c}\times n_{g}}$, with $n_{c}$ indicating the number of cells in the data and $n_{g}$ indicating the number of genes in the data, and mIHC spatial features denoted as $X^{\left(m\right)}$, which has the dimensions ${\mathbb{R}}^{n_{r}\times d_{m}}$, with $n_{r}$ representing the number of regions represented in the data and $d_{m}$ representing the number of spatial feature dimensions that exist. Clinical variables are represented by the matrix $X^{\left(c\right)}$, which has the dimensions ${\mathbb{R}}^{d_{c}}$.

The single-cell encoder uses a graph attention network architecture [23] to understand how cells interact with one another based upon their transcriptional profiles. The cell–gene expression matrix is converted into a *k*-nearest neighbor graph of the form $G=\left(V,\;E\right)$, where V denotes the set of nodes representing cells and E denotes the set of edges connecting cells that have similar transcriptional profiles. Using this graph attention architecture, the following steps are computed:

$$\alpha_{\mathrm{ij}}=\frac{\exp\left(\text{LeakyReLU}\left(a^{T}\left[Wh_{i}\;∥\;Wh_{j}\right]\right)\right)}{\sum_{k\in {𝒩}_{i}}\exp\left(\text{LeakyReLU}\left(a^{T}\left[Wh_{i}\;∥\;Wh_{k}\right]\right)\right)}$$ (1)

where $h_{i}$ denotes the feature vector for cell i, W is a learnable weight matrix, a is the attention weight vector, and $N_{i}$ represents the neighborhood of cell i. The updated cell representations are computed as

$$h_{i}^{\prime }=\sigma \left(\sum_{j\in {𝒩}_{i}}\alpha_{ij}Wh_{j}\right)$$ (2)

**Fig. 3.**
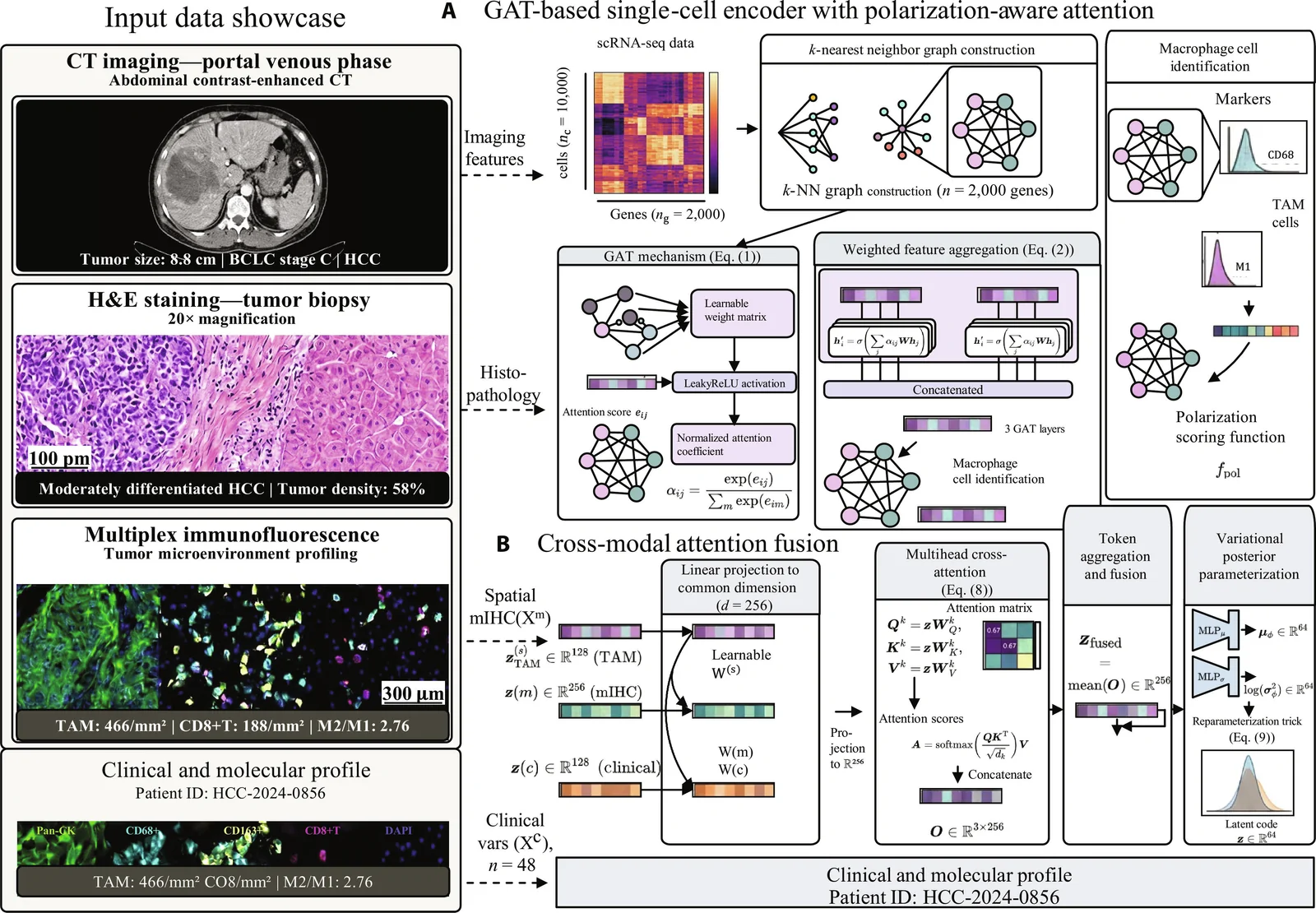
The figure provides a detailed view of the architecture for the single-cell encoder and the cross-modality attention fusion modules. (A) The encoder consists of 4 stages, starting with building a *k*-nearest neighbor graph on the gene expression information ($X^{\left(s\right)}\in {\mathbb{R}}^{n_{c}\times n_{g}}$). The second stage involves calculating the attention coefficients ($\alpha_{\mathrm{ij}}$) using the graph attention network (GAT) method (see Eq. (1)), the third stage involves aggregating data from 3 layers of GAT using weighted features to update a cell’s representation ($h_{i}^{\prime }\in {\mathbb{R}}^{128}$) (see Eq. (2)), and finally filtering out CD68+ macrophage cells and combining information from these cells with the polarization information using attention scored by the function $f_{\mathrm{pol}}$ that assigns the extent of macrophage polarization toward M1 versus M2 phenotypes ($-1\leq s_{i}\leq +1$) (see Eq. (3)) along with the single-cell representation to create a tumor-associated macrophage (TAM)-specific representation of the macrophage ($z_{\mathrm{TAM}}^{\left(s\right)}\in {\mathbb{R}}^{128}$). The left side shows the type of data that was inputted, such as computerized tomography (CT) scans, hematoxylin and eosin (H&E)-stained tissue sections of 20× magnification, and multiplex immunofluorescence stainings (pan-cytokeratin [pan-CK], CD68, CD163, CD8+ T, and 4′,6-diamidino-2-phenylindole [DAPI]) for a representative hepatocellular carcinoma (HCC) patient (ID: HCC-2024-0856). (B) The fusion modules integrate information from each modality-specific representation ($z_{\mathrm{TAM}}^{\left(s\right)}$, $z^{\left(m\right)}$, and $z^{\left(c\right)}$) by first mapping them into a single, shared 256-dimensional space using learnable weight matrices ($W^{\left(s\right)}$, $W^{\left(m\right)}$, and $W^{\left(c\right)}$) and then computing attention scores using the cross-attention mechanism (see Eq. (8)) as described in the previous section to obtain a single fused output ($O\in {\mathbb{R}}^{3\times 256}$). The fused output is used to calculate the parameters for the posterior ($\mu_{\phi }\in {\mathbb{R}}^{64}$, $\log\left(\sigma_{\phi }^{2}\right)\in {\mathbb{R}}^{64}$), where $\mu_{\phi }$ and $\sigma_{\phi }$ are then used to sample $z∼N\left(\mu_{\phi },\;\mathrm{diag}\left(\sigma_{\phi }^{2}\right)\right)$ through the reparameterization trick. Scale bars: 100 μm (histopathology) and 300 μm (multiplex immunofluorescence).

Fig. 3 provides a complete visual representation of how to encode a single cell in full detail, including processing the input data before use, creating the *k*-nearest neighbor graph, calculating attention via graph attention networks (with derived weights), and aggregating attention matrices while also accounting for any polarized TAMs or other types of asymmetrical distribution between cells.

TAM-specific representations are aggregated using a polarization-aware attention mechanism:

$$\begin{array}{c} z_{\mathrm{TAM}}^{\left(s\right)}=\sum_{i\in M}\beta_{i}h_{i}^{\prime }\\ \beta_{i}=\frac{\exp\left(f_{\mathrm{pol}}\left(h_{i}^{\prime }\right)\right)}{\sum_{j\in M}\exp\left(f_{\mathrm{pol}}\left(h_{i}^{\prime }\right)\right)} \end{array}$$ (3)

where M refers to a collection of macrophage cells and the function $f_{\mathrm{pol}}$ is an artificial-neural-network-based scoring function for classifying the polarization levels.

The spatial encoder applies a hierarchical approach to space, which encodes local spatial patterns into a regional feature vector for processing. The encoded regional feature vector $X^{\left(m\right)}$ is generated by a local (convolutional) layer with the support of a pooling layer applying the same convolutional kernel that generates local features within the same pool.

$$F^{\left(l\right)}=\text{ReLU}\left(\text{Conv}2D\left(X^{\left(m\right)};\;\theta_{\text{conv}}\right)\right)$$ (4)

Global spatial context is then integrated using a spatial transformer module [24]:

$$Q=F^{\left(\text{l}\right)}W_{Q},\;K=F^{\left(\text{l}\right)}W_{K},\;V=F^{\left(l\right)}W_{V}$$ (5)

$$z^{\left(m\right)}=\text{softmax}\left(\frac{QK^{T}}{\sqrt{d_{k}}}\right)V$$ (6)

The clinical encoder is a multilayer perceptron that processes tabular clinical features:

$$z^{\left(c\right)}=\mathrm{MLP}\left(X^{\left(c\right)};\;\theta_{\text{clin}}\right)$$ (7)

Modal-specific representations are fused through a cross-modal attention mechanism that learns complementary information across data sources:

$$\begin{array}{c} z_{\text{fused}}=\text{CrossAttn}\left(z_{\mathrm{TAM}}^{\left(s\right)},\;z^{\left(m\right)},\;z^{\left(c\right)}\right)\\ \;=\mathrm{MHA}\left(\left[z_{\mathrm{TAM}}^{\left(s\right)};\;z^{\left(m\right)};\;z^{\left(m\right)}\right]\right) \end{array}$$ (8)

where MHA denotes multihead attention.

The fused representation parameterizes the approximate posterior distribution:

$$q_{\phi }\left(z|X\right)=N\left(z;\mu_{\phi }\left(z_{\text{fused}}\right),\;\mathrm{diag}\left(\sigma_{\phi }^{2}\left(z_{\text{fused}}\right)\right)\right)$$ (9)

Let $\mu_{\phi }$ and $\sigma_{\phi }$ represent the outputs of the neural network. The structure of the latent space was guided by the Kullback–Leibler-weighted VAE formulation combined with hierarchical priors so as to produce clinically interpretable representations. A tunable constant β was used in the β-VAE framework [25,26] to create a trade-off between the reconstruction fidelity of the data and the regularization on the latent representation; through hyperparameter optimization, β was established at 0.1 to optimise for most predictive capability while generating a meaningful latent representation.

#### Temporal dynamics module

The TDM describes how immune states evolve over time and as a function of therapeutic interventions. By providing an initial latent state $z_{0}$ and a sequence of treatment actions $a_{1},\ldots ,a_{T}$, we allow the module to predict what latent states $z_{1},\ldots ,z_{T}$ will occur in the future and the trajectory of those states.

This module’s architecture is based on a transformer decoder [27] that implements causal masking to prevent attending to future time points; it is further modified by concatenating embedded treatment action information with the latent immune state sequence.

$$H_{0}=\left[z_{0}+e_{a_{1}};\ldots ;\;z_{T-1}+e_{a_{T}}\right]$$ (10)

where $e_{a_{t}}$ denotes the learned embedding for action $a_{t}$.

The transformer dynamics are computed as

$$\begin{array}{c} H^{\left(l\right)}=\text{LayerNorm}\left(H^{\left(l-1\right)}+\text{MaskedMHA}\left(H^{\left(l-1\right)}\right)\right)\\ H^{\left(l\right)}=\text{LayerNorm}\left(H^{\left(l\right)}+\mathrm{FFN}\left(H^{\left(l\right)}\right)\right) \end{array}$$ (11)

The predicted latent states are obtained through a projection layer:

$${\hat{z}}_{t}=W_{\text{out}}H_{t}^{\left(L\right)}+b_{\text{out}}$$ (12)

To capture the stochastic nature of immune dynamics, the temporal module outputs parameters of a predictive distribution rather than point estimates:

$$p_{\theta }\left(z_{t+1}|z_{\leq t},\;a_{\leq t+1}\right)=𝒩\;({\hat{\mu }}_{t},\;{\hat{\Sigma }}_{t})$$ (13)

The dynamics module is trained to minimize the negative log-likelihood of observed state sequences while maintaining consistency with the learned latent representations:

$$\begin{array}{c} L_{\mathrm{dyn}}=-\sum_{t=1}^{T}\log\;p_{\theta }\left(z_{t}|z_{<t},\;a_{\leq t}\right)\\ \;+\lambda_{\text{cons}}{\left\|{\hat{z}}_{t}-z_{t}\right\|}_{2}^{2} \end{array}$$ (14)

#### Treatment optimization layer

Using the learned world model as a basis, the treatment optimization layer [28] will utilize the world model to develop and define a number of customized treatment plans. Given the patient’s starting immune status, denoted by the vector $z_{0}$, we will find the sequence of actions $a_{1}^{*},\ldots ,a_{T}^{*}$ that would yield the maximum expected total reward over the planning horizon.

$${\left\{a_{t}^{*}\right\}}_{t=1}^{T}=\arg\max_{\left\{a_{t}\right\}}\;E\;\left[\sum_{t=1}^{T}\gamma^{t-1}R(z_{t},\;a_{t})\right]$$ (15)

where γ is a discount factor and $R\left(z_{t},\;a_{t}\right)$ is a reward function encoding clinical objectives.

The reward function is designed to capture multiple aspects of treatment efficacy:

$$\begin{array}{c} R(z_{t},a_{t})=w_{1}\cdot R_{\text{response}}\left(z_{t}\right)+w_{2}\cdot R_{\mathrm{TAM}}\left(z_{t}\right)\\ \;+w_{3}\cdot R_{\text{toxicity}}\left(a_{t}\right)+w_{4}\cdot R_{\text{survival}}\left(z_{t}\right) \end{array}$$ (16)

where $R_{\text{response}}$ indicates the expected tumor response, $R_{\mathrm{TAM}}$ reflects the favorable polarization of TAMs, $R_{\text{toxicity}}$ indicates potential toxicity/excess treatment associated adverse events, and $R_{\text{survival}}$ accounts for estimated survival probabilities. Detailed definitions of each reward component, the treatment action space ($\left|A\right|=847$ clinically validated regimens), and the uncertainty quantification procedure are provided in Section S6.

Model-predictive control (MPC) is used to optimize the treatment with cross-entropy method sampling [29] (Algorithm 1). At each decision time step, we random sample N potential actions based on the proposal distribution, predict each candidate’s expected returns using the world model, and iterate to improve the proposals based on high-expectation regions:

$$\begin{array}{c} {\left\{a_{1:T}^{\left(i\right)}\right\}}_{i=1}^{N}∼p_{\text{proposal}}\left(a_{1:T}\right)\\ \;J^{\left(i\right)}=\sum_{t=1}^{T}\gamma^{t-1}R({\hat{z}}_{t}^{\left(i\right)},\;a_{t}^{\left(i\right)}) \end{array}$$ (17)

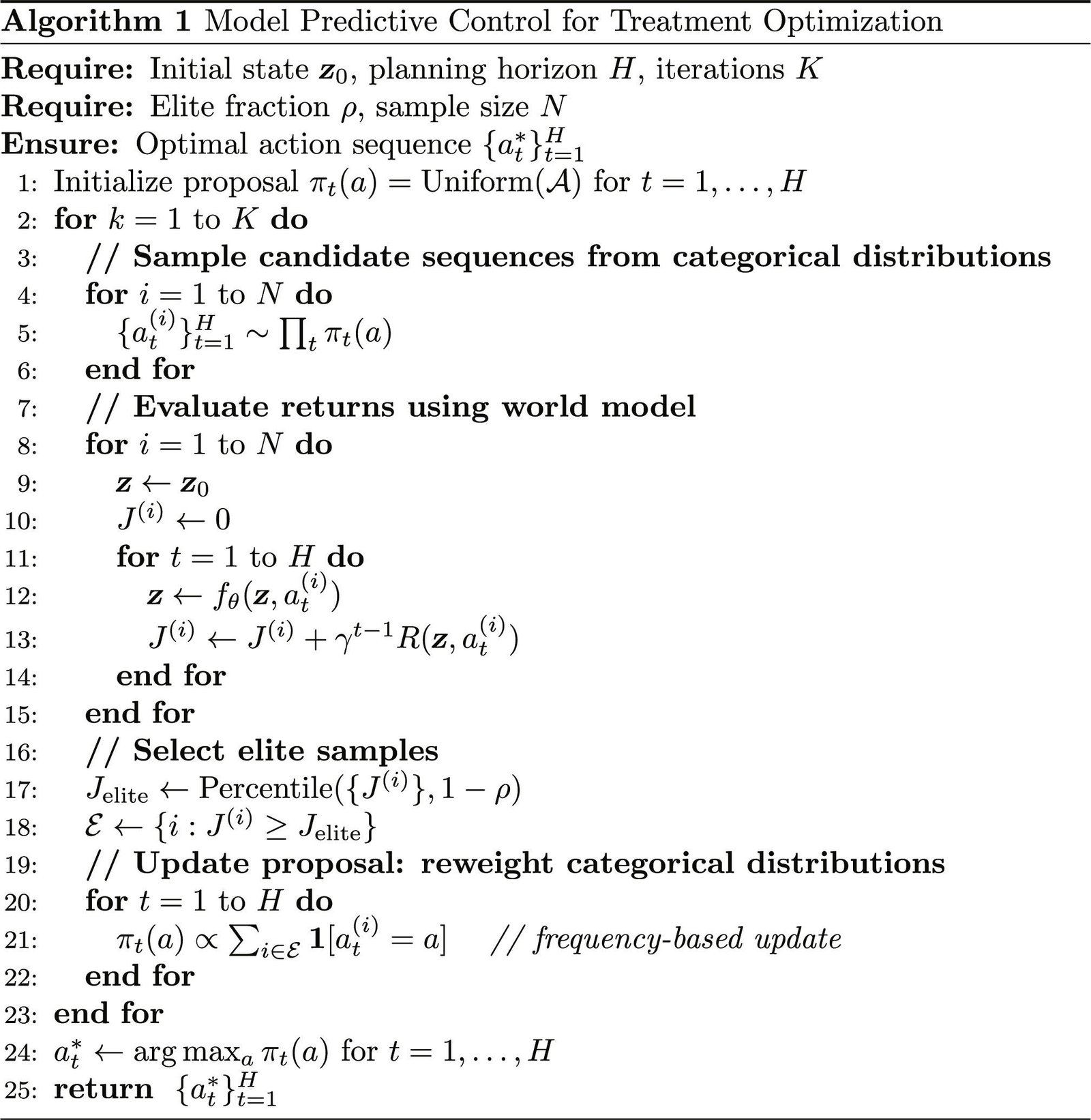


The proposal distribution is updated by fitting to the elite samples with the highest returns:

$$p_{\text{proposal}}\leftarrow \mathrm{fit}\left(\left\{a_{1:T}^{\left(i\right)}:J^{\left(i\right)}\geq J_{\text{elite}}\right\}\right)$$ (18)

### Training procedure

The complete GI-ImmunoWorld model is trained end to end using a composite objective function that combines reconstruction, regularization, and dynamics losses:

Ltotal=Lrecon+βLKL+λdynLdyn+λpredLpred (19)

The reconstruction loss ensures faithful encoding of multimodal inputs:

$$\begin{array}{c} L_{\text{recon}}=E_{q_{\phi }\left(z|X\right)}\left[-\log\;p_{\psi }\left(X^{\left(s\right)}|z\right)\right.\\ \left.\;-\log\;p_{\psi }\left(X^{\left(m\right)}|z\right)-\log\;p_{\psi }\left(X^{\left(c\right)}|z\right)\right] \end{array}$$ (20)

The Kullback–Leibler divergence regularization encourages structured latent representations:

$$L_{\mathrm{KL}}=D_{\mathrm{KL}}\left(q_{\phi }\left(z|X\right)\;\;∥\;\;p\left(z\right)\right)$$ (21)

The prediction loss supervises response and survival outcomes:

$$\begin{array}{c} \begin{array}{c} L_{\text{pred}}=-\sum_{i}\left[y_{i}\log\;{\hat{y}}_{i}+\left(1-y_{i}\right)\log\left(1-{\hat{y}}_{i}\right)\right]\\ \;+\sum_{i}L_{\mathrm{Cox}}\left(z_{i},\;t_{i},\;\delta_{i}\right) \end{array} \end{array}$$ (22)

where $L_{\mathrm{Cox}}$ denotes the Cox partial likelihood loss for survival modeling.

**Fig. 4.**
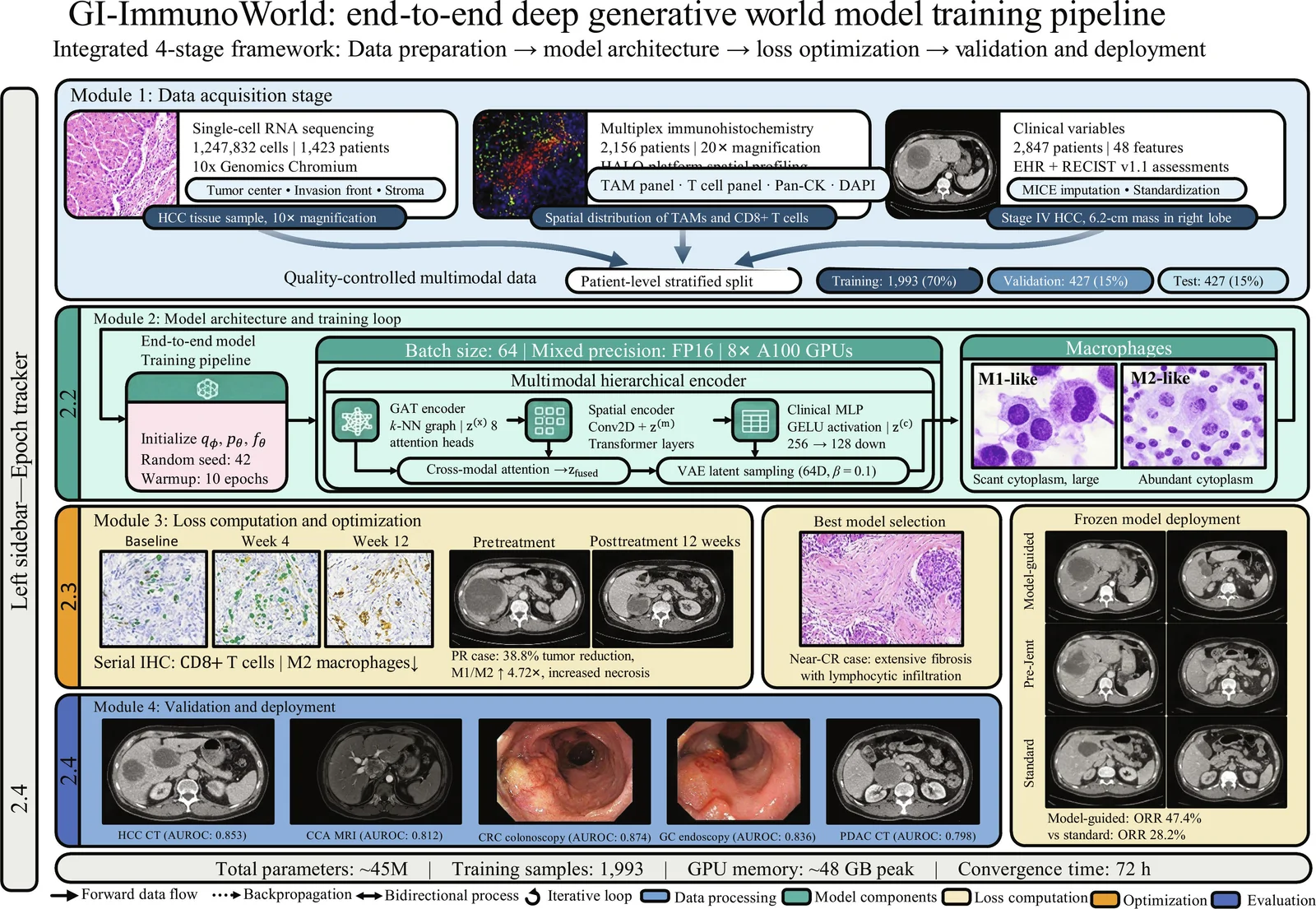
The GI-ImmunoWorld end-to-end training pipeline is built around a standard 4-stage training framework. Module 1 provides the context for the creation of the pipeline through the acquisition and quality control of the 3 different types of data used in the pipeline (single-cell RNA sequencing, multiplex immunohistochemistry examining multiple cell types on multiple slides, and clinical variables). Module 2 describes how the model architecture and training loop are created. To do this, it describes how to create a hierarchical multimodal encoder that includes 3 different encoders (single-cell RNA sequencing, multiplex immunohistochemistry, and clinical variables). The 3 encoders are combined via cross-modal attention using a variational autoencoder (VAE) latent space to integrate the 3 encoder outputs before being used as inputs to the model. Module 3 details how to create and use the loss function to train the model. In this section, we list the key steps to monitor how well we are training the model, through reports and examples from individual patients. Module 4 describes how the model will be validated and deployed for the treatment of patients. In this module, we provide examples of how the model was tested and deployed across 5 different tumor types. These included tumor-specific area under the receiver operating characteristic curve (AUROC) for each of the 5 tumor types. The bottom part of this module includes representative images showing how patients who were treated with model-guided therapy have had marked increases in the number of CD8+ T cells and a decrease in the number of M2 macrophages in comparison with patients who were treated with standard care. The left side of this graphic shows the elapsed time in terms of the progression of training through initialization, warmup, loss stabilization, mid-training, convergence, and completion and shows that the total training of the model will take approximately 200 epochs. The total number of parameters in the model is approximately 45 million. The total amount of memory used on a graphics processing unit (GPU) will be approximately 48 GB, and the model’s total training time is approximately 72 h.

We utilized the AdamW optimization technique with decoupled L2 regularization (0.01), a learning rate of 1 × 10^−4^, and a cosine-based learning rate decay schedule during the 10 warming epochs and then trained our models using a batch size of 64 and a maximum gradient clip at 1.0 for a total of 200 epochs on 8 NVIDIA A100 graphics processing units (GPUs) with mixed-precision training enabled (Tables S2 and S3). We implemented early stopping by monitoring the lowest recorded validation loss across the entire training period and implemented an early stopping criterion after allowing for 20 epochs with no recorded declines in the validation loss. We optimized hyperparameters using Bayesian hyperparameter tuning on the withheld validation dataset that represented 15% of patients’ samples; thus, all samples/times were separated by patient, thus avoiding any potential leakage of data from one dataset to another (Table S4). The complete picture of the training pipeline is illustrated in Fig. 4, which includes all phases of the end-to-end training, including acquiring data, creating and optimizing the model architecture, loss function, and deployment of the trained models.

### Evaluation metrics and statistical analysis

Multiple metrics related to relevant clinical endpoints were used to evaluate model performance. For the prediction of the response to a treatment, we calculated AUROC, the area under the precision–recall curve, sensitivity, specificity, and F1 scores. To evaluate survival prediction, we calculated the concordance index (C-index); AUROC at 6, 12, and 24 months (time dependent); and integrated Brier score.

Statistical significance was determined using 2-sided tests with a significance threshold of α=0.05. Methods were compared using the DeLong test to determine differences in AUROC and via the log-rank test for comparisons of survival. Confidence intervals (CIs) were calculated via 1,000 bootstrap resamples. For the main comparison between GI-ImmunoWorld and several baseline models, the Benjamini–Hochberg procedure for controlling the false discovery rate was applied, with all statistically significant differences remaining statistically significant after correction (adjusted P<0.05). Detailed test statistics, raw *P* values and adjusted *P* values, and their corresponding CIs can be found in the supplementary tables. For clarity in the notation in Algorithm 2, the survival data $\left(t_{i},\;\delta_{i}\right)$, or time and event indicator, are collectively referred to as $t_{i}$.


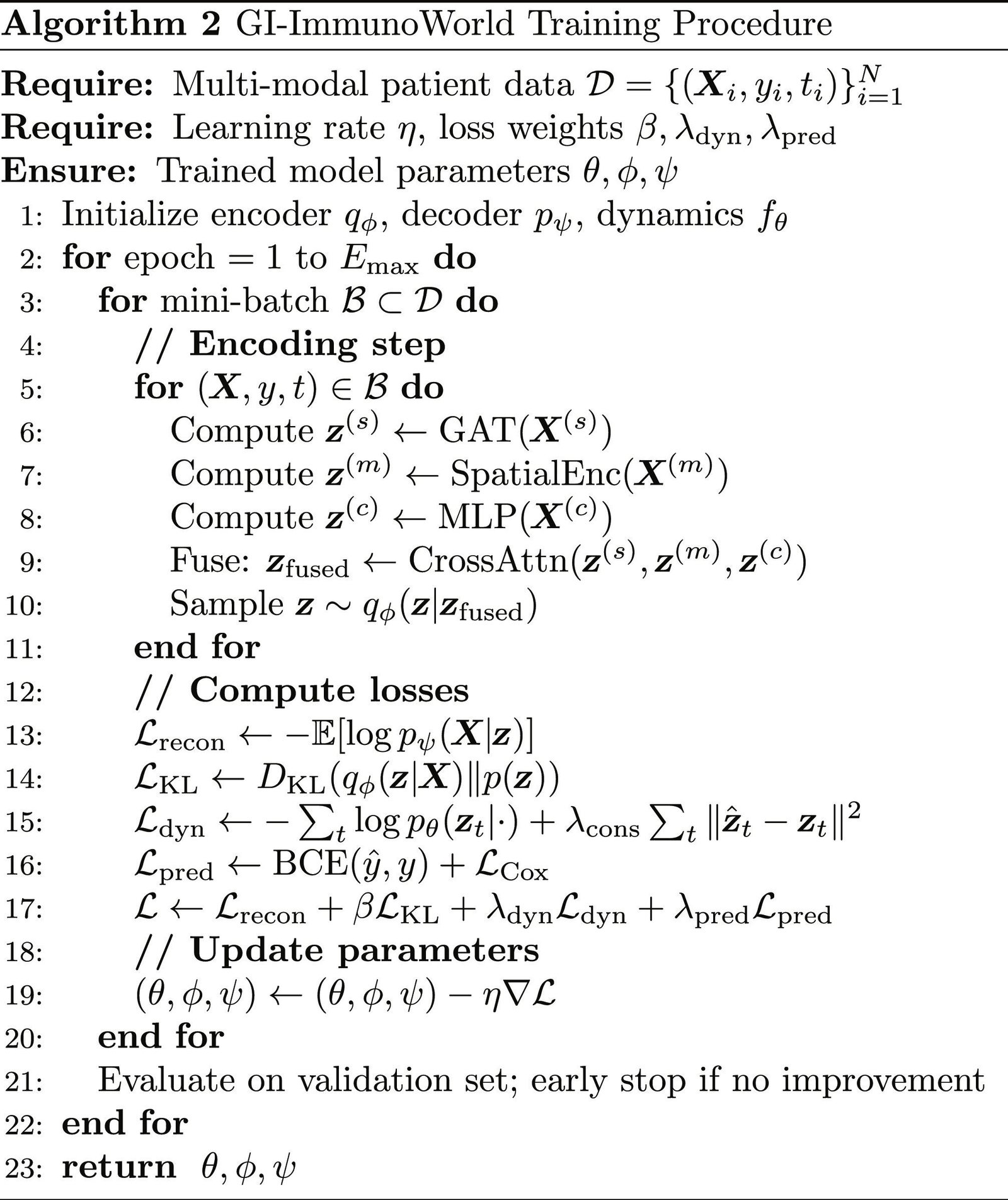


## Results

### Patient characteristics and cohort description

The study participants consisted of 2,847 individuals diagnosed with hepatobiliary–intestinal tumors who were treated with immunotherapies between January 2018 and December 2023 (the historical dataset for development of predictive models). One hundred fifty-six patients enrolled in studies conducted from January 2024 through March 2025 made up the futuristic sample group to assess predictive model performance. The historical patient dataset was stratified and separated based on tumor type and treatment response to form 3 distinct datasets: training (*N* = 1,993, 70%), validation (*N* = 427, 15%), and testing (*N* = 427, 15%). Table 1 provides descriptive information on all study subjects.

**Table 1.** Baseline characteristics of the study cohort stratified by tumor type

Tumor type (cases)	HCC (*n* = 684)	CCA (*n* = 412)	CRC (*n* = 892)	GC (*n* = 523)	PDAC (*n* = 336)
Characteristics					
Age, median (IQR)	62 (54–69)	65 (57–72)	59 (51–67)	63 (55–70)	66 (58–73)
Male/%	74.2	52.4	56.8	64.1	53.9
ECOG PS 0–1/%	78.5	71.8	82.3	76.4	68.2
Stage IV/%	68.4	72.1	54.3	61.8	78.6
Prior systemic Tx/%	45.2	38.6	52.1	48.7	34.8
PD-L1 ≥ 1%/%	42.8	35.2	48.6	39.4	28.3
MSI-H/dMMR/%	3.2	2.4	14.8	8.6	1.8
Treatment regimen					
Anti-PD-1/PD-L1 mono	38.2	42.5	35.6	40.2	45.8
ICI + TKI	48.5	32.8	28.4	35.2	24.1
ICI + chemo	13.3	24.7	36.0	24.6	30.1
Response (RECIST)					
CR/PR/%	26.8	18.4	32.5	24.1	14.6
SD/%	34.2	28.6	31.8	32.4	26.8
PD/%	39.0	53.0	35.7	43.5	58.6
mPFS/months	6.8	5.2	8.4	6.1	4.3
mOS/months	14.2	11.8	18.6	13.4	9.2

HCC, hepatocellular carcinoma; CCA, cholangiocarcinoma; CRC, colorectal cancer, GC, gastric cancer, PDAC, pancreatic ductal adenocarcinoma; IQR, interquartile range; ECOG PS, Eastern Clinical Oncology Group performance status; MSI-H/dMMR, microsatellite instability-high/mismatch repair deficient; PD-L1, programmed death-ligand 1; PD-1, programmed cell death protein 1; ICI, immune checkpoint inhibitor; TKI, tyrosine kinase inhibitor; RECIST, response evaluation criteria in solid tumors; CR/PR, complete response/partial response; SD, stable disease; PD, progressive disease; mPFS, median progression-free survival; mOS, median overall survival

The median age of participants in this study was 62, while the interquartile range was 54 to 70. The cohort was predominantly male (60.8%), with the majority having good performance status (Eastern Cooperative Oncology Group [ECOG] 0 to 1: 76.4%) and advanced-stage disease (stage IV: 66.1%). The ORR was calculated using the weighted average, with the lowest ORR of 14.6% for PDAC and the highest one of 32.5% for CRC. The median PFS was 6.4 months, and the median OS was 13.8 months.

### Single-cell and spatial characterization of the immune landscape

An investigation into the TME using scRNA-seq data containing a total of 1,247,832 single cells resulted in the generation of comprehensive maps of the TME according to tumor histology. Unsupervised clustering analysis yielded information about 28 unique cell types, including 6 subtypes of macrophages with distinct polarization states. Table S5 shows the cellular compositions for each tumor type based on the scRNA-seq data collected.

**Fig. 5.**
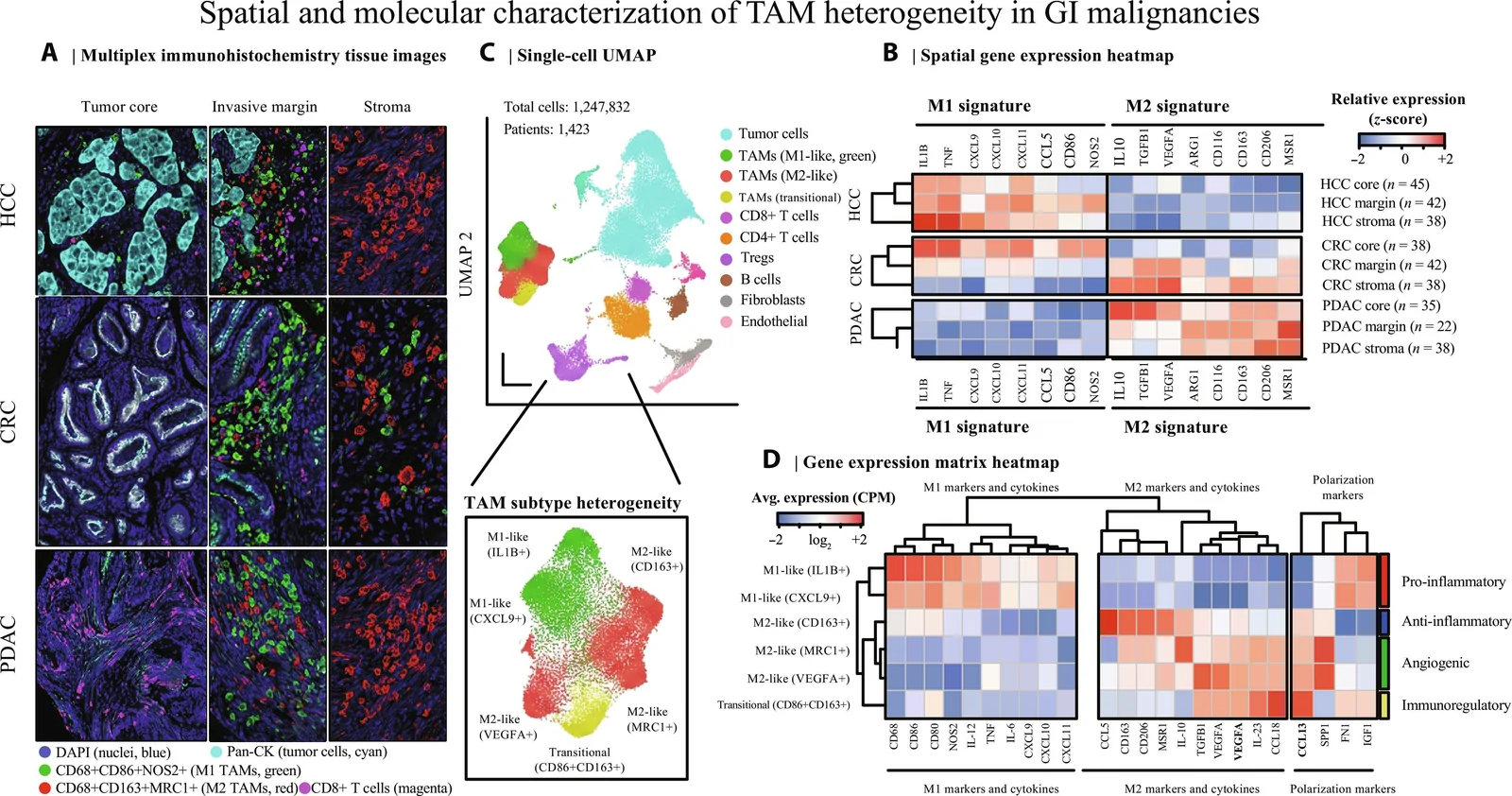
Spatial and molecular characterization of tumor-associated macrophage (TAM) heterogeneity in gastrointestinal tumors. (A) Representative multiplex immunohistochemistry (mIHC) images illustrating the spatial arrangements of tumor cells (pan-CK+, cyan), M1-like TAMs (CD68+CD86+NOS2+, green), M2-like TAMs (CD68+CD163+MRC1+, red), and CD8+ T cells (magenta) found in the core of the tumors, at the invasive front, and in the stroma, among 3 types of gastrointestinal (GI) cancers, hepatocellular carcinoma (HCC), colorectal cancer (CRC), and pancreatic ductal adenocarcinoma (PDAC). Nuclei were counterstained with 2′,6-diamidino-2-phenylindole (DAPI; blue). Scale bars, 100 μm. (B) Heatmap demonstrating relative levels of expression (*z*-score) of M1 and M2 markers across the 3 GI cancers and the different spatial regions. *N* represents HCC samples *n* = 125, CRC *n* = 118, and PDAC *n* = 95. (C) Uniform manifold approximation and projection (UMAP) presentation of 1,247,832 cells from 1,423 patients across 5 GI cancer types reveals 10 major cell types. Inset: Clusters of TAM subtypes exhibiting different states of polarization. (D) Heatmap of the average gene expression levels (log_2_ CPM) of important M1 and M2 markers, cytokines, and transcription factors that regulate cell polarization of the 6 separate TAM subsets formed from single-cell analysis and their associated functional classification.

The way that TAM populations differ from one another in terms of space and their different styles of existence based on what kind of tumor type they are associated with is shown in Fig. 5.

Clinical outcomes were significantly associated with the M2/M1 ratio across every tumor type evaluated. The M2/M1 ratio was associated with significantly decreased levels of response and shorter OS among patients with M2/M1 ratios greater than the median (18.4% vs 31.2% for response, P<0.001; 5.2 months vs 8.1 months for median PFS, P<0.001). Analysis of the spatial relationships between TAMs and CD8+ T cells indicated that assessing the degree of proximity of CD8+ T cells to TAMs is potentially related to the response; that is, those patients who responded demonstrated greater degrees of spatial interaction (mean of 0.68 vs 0.42 among responders and nonresponders, respectively, P<0.001).

### Immunotherapy response prediction performance

Compared to other established immunotherapy response prediction methods—such as clinical biomarkers (PD-L1, tumor mutational burden, and microsatellite instability), tumor gene expression profiles (Tumor Inflammation Signature [30], TIDE [31], and ImmuneCells.Sig [32]), and state-of-the-art machine learning tools such as DeepTIL [33], MCAT [34], PORPOISE [35], TransMIL [36], SurvPath [37], and CONCH [38]—GI-ImmunoWorld had a significant advantage because most baseline methods’ predictions were derived solely from histopathological images without considering other relevant clinical factors. However, GI-ImmunoWorld incorporated scRNA-seq data whenever available. Consequently, this comparison reflects the potential for clinical practice to utilize more complex multimodal assays rather than simply being a fair evaluation of different predictive algorithms using the same inputs. GI-ImmunoWorld’s predictive capability based upon testing out-of-sample cases is shown in Table 2.

**Table 2.** Comparative analysis of predictive performance across clinical biomarkers, deep learning methods, and GI-ImmunoWorld for immunotherapy response prediction. Note that input modalities differ across methods; a controlled comparison using identical inputs is provided in Table S8.

Method	AUROC	AUPRC	Accuracy	Sensitivity	Specificity	F1
Clinical biomarkers (all test patients, *n* = 427)						
PD-L1 CPS ≥ 1	0.584	0.312	0.562	0.486	0.612	0.418
TMB ≥ 10 mut/Mb	0.612	0.348	0.578	0.524	0.602	0.445
MSI-H/dMMR	0.538	0.286	0.524	0.418	0.582	0.362
Combined biomarkers	0.692	0.426	0.638	0.582	0.668	0.512
Gene expression signatures (scRNA-seq subset, *n* = 214)						
TIS [30]	0.658	0.394	0.612	0.548	0.652	0.482
TIDE [31]	0.672	0.408	0.624	0.562	0.658	0.496
ImmuneCells.Sig [32]	0.648	0.386	0.602	0.538	0.642	0.468
Deep learning methods (WSI + clinical, *n* = 427)						
DeepTIL [33]	0.724	0.468	0.672	0.618	0.702	0.562
MCAT [34]	0.756	0.512	0.698	0.642	0.728	0.594
PORPOISE [35]	0.768	0.528	0.712	0.658	0.742	0.612
TransMIL [36]	0.742	0.494	0.686	0.628	0.718	0.578
SurvPath [37]	0.774	0.536	0.718	0.664	0.746	0.618
CONCH [38]	0.786	0.554	0.728	0.678	0.756	0.634
GI-ImmunoWorld (scRNA + mIHC + clinical, *n* = 427)						
Full model	0.847	0.624	0.782	0.738	0.806	0.712
W/o temporal	0.812	0.578	0.748	0.694	0.778	0.668
W/o multimodal	0.794	0.556	0.732	0.676	0.764	0.642
W/o polarization	0.824	0.596	0.762	0.712	0.790	0.686

AUROC, area under the receiver operating characteristic curve; AUPRC, area under the precision–recall curve; PD-L1, programmed death-ligand 1; CPS, combined positive score; TMB, tumor mutational burden; MSI-H/dMMR, microsatellite instability-high/mismatch repair deficient; scRNA-seq, single-cell RNA sequencing; TIS, Tumor Inflammation Signature; WSI, whole-slide imaging; mIHC, multiplex immunohistochemistry

GI-ImmunoWorld obtained an AUROC value of 0.847 (95% CI: 0.812 to 0.878), which represents a statistically significant increase compared to the highest-performing CONCH [38] baseline (AUROC 0.786, ΔAUROC = 0.061, P<0.001 by DeLong’s test) and the other baseline evaluation metrics, with the most significant improvement in sensitivity (0.738 vs 0.678) indicating enhanced performance in identifying responders to treatment. All of the architectural components (as demonstrated through the ablation studies) contributed to the performance of GI-ImmunoWorld with the TDM having the largest individual contribution (ΔAUROC = 0.035 upon removal) (Table S6). Five-fold cross-validation confirmed the stability of these results (AUROC mean = 0.847, SD = 0.007; Table S7).

The second goal of this study was to separate the contribution of performance improvement from the introduction of new input modalities and architectural innovations within GI-ImmunoWorld based on the input modalities.

To do this, we developed a histopathology version of GI-ImmunoWorld using mIHC-derived features combined with clinical variables without scRNA-seq data inputs. The AUROC for this reduced-input variant was 0.794 (95% CI: 0.758 to 0.830; Table S8). Latent dimension correlations with established immune markers are detailed in Table S9. As a result, the additional gain from the full multimodal model (ΔAUROC = 0.053) reflects the benefit associated with including information about single-cell molecular profiles. We note that the primary comparison in Table 2 shows that the benefits associated with comprehensive profiling reflect clinically achievable performance not necessarily algorithmic superiority to those models that are developed using identical input modalities. The most appropriate means to measure the contribution of each of the architectural components to the model performance is through ablation studies (without temporal: −0.035; without multimodal: −0.053; without polarization: −0.023) (Table S6).

To evaluate if GI-ImmunoWorld’s performance was generalizable across various tumor types, GI-ImmunoWorld was also evaluated based on the individual tumor type performance. The results from the individual tumor type evaluations are presented in Table S10.

The world model exhibited strong accuracy in all tumor types with AUROC ranging from 0.798 (PDAC) to 0.874 (CRC). In addition, the world model provided the most improved results (compared to benchmarks) for PDAC (ΔAUROC = 0.064 versus CONCH), a tumor type with an immunosuppressive TME and historically low response to immunotherapies. These results suggest that the architecture of the world model is particularly suited for the capture of complex immune dynamics occurring in very difficult-to-treat TMEs.

### Survival prediction and prognostication

Beyond response prediction, GI-ImmunoWorld was evaluated for survival prognostication. Table 3 presents the survival prediction performance, comparing GI-ImmunoWorld against Cox-PH, DeepSurv [39], SALMON [40], SurvPath [37], MCAT [34], PORPOISE [35], and CONCH [38].

**Table 3.** Survival prediction performance comparison

Method	C-index	AUROC@12m	AUROC@24m	IBS
Cox-PH (clinical)	0.612	0.624	0.608	0.218
DeepSurv [39]	0.658	0.672	0.654	0.194
SALMON [40]	0.684	0.698	0.682	0.178
SurvPath [37]	0.712	0.728	0.706	0.162
MCAT [34]	0.698	0.714	0.692	0.172
PORPOISE [35]	0.708	0.722	0.698	0.168
CONCH [38]	0.724	0.738	0.718	0.156
GI-ImmunoWorld	0.786	0.802	0.778	0.128

C-index, concordance index; AUROC, area under the receiver operating characteristic curve; AUROC@12m, AUROC at 12 months; AUROC@24m, AUROC at 24 months; IBS, integrated Brier score

The concordance index for GI-ImmunoWorld was 0.786 (95% CI: 0.752 to 0.818), which is statistically significantly better than CONCH’s C-index (0.724) (P<0.001). The time-dependent AUROC at 12 months was 0.802, indicating strong discriminating ability at 12 months for medium-term prognosis. The integrated Brier score of 0.128 for this model indicates excellent calibration of survival probability estimates for patients.

Risk stratification of patients based on model prediction results yielded clinically distinct prognostic groups. Patients in the high-risk tertile had a median OS of 8.4 months, while patients in the low-risk tertile had a median OS of 22.6 months (hazard ratio [HR] = 3.42; 95% CI: 2.68 to 4.36; P<0.001).

### Latent space analysis and interpretability

The identified latent representations were evaluated for their biological meanings and clinical implications. The 64-dimensional latent space was visualized through uniform manifold approximation and projection dimensionality reduction analysis, resulting in the formation of unique clusters representing TAM polarization states and treatment response phenotypes.

The correlations between the most informative latent dimensions and previously established immune markers are reported in Table S9.

The first latent dimension (referred to as $z_{1}$) has a strong positive correlation with the M1 macrophage score (*r* = 0.72; 95% CI: 0.67 to 0.77) and a negative correlation with the M2 score (*r* = −0.68; 95% CI: −0.73 to −0.62), allowing it to effectively capture the M1/M2 polarization axis. The third latent dimension (referred to as $z_{3}$) demonstrates a strong association with CD8+ T cell infiltration (*r* = 0.82; 95% CI: 0.78 to 0.86), indicating the cytotoxic immune compartment. In conclusion, the architecture of the VAE successfully provides disentangled representations of specific immunological elements, which means that there is no ambiguity as to what they represent, without needing to provide any kind of explicit supervision.

### Temporal dynamics validation

The temporal dynamics model was tested against longitudinal data from a cohort of patients that had multiple biopsy samples (*n* = 89) to validate the model by comparing expected immunological trajectories to observed immunological trajectories. The accuracy of the immunological trajectory predictions can be found in Table S11.

A high correlation (*r* = 0.86) was found between the predicted latent trajectory of the model and the observed latent trajectory with an $R^{2}$ statistic indicating that 72% of the variation in immune system dynamics could be explained by the world model. Among all predictors used in the model, interferon-γ (IFN-γ) showed the highest correlation (*r* = 0.88), and the density of other T cells also demonstrated a strong correlation (*r* = 0.86); both were identified as the predictors with the greatest potential clinical relevance for response to immunotherapy.

The results from temporal predictions identified opportunities for repolarizing macrophages. Repolarization windows were operationally defined as time intervals during which the TDM predicted a >0.3 shift in the M1/M2 latent dimension ($z_{1}$) toward the M1 direction within the subsequent 4-week period. Patients who initiated immunotherapy during these predicted windows showed significantly higher response rates (42.8% vs 21.4%, P=0.008).

### Treatment optimization and clinical validation

In an exploratory prospective deployment study, we examined the treatment optimization capabilities of GI-ImmunoWorld. According to the methods section, this study was conducted on the “discordant cohort” (i.e., patients whose model recommendation and treating physician’s initial standard of care plan differed) in which treatment decisions by physicians were classified as observational and nonrandomized. The discordant cohort included 156 patients of the total 312 patients participating in the study prospectively. In the discordant cohort, 78 patients received treatment according to the model’s recommendation (model-guided group), whereas the remaining 78 received the original standard of care as recommended by their physician (standard group). The clinical outcomes are presented in Table 4.

**Table 4.** Exploratory, hypothesis-generating analysis of the discordant cohort (*n* = 156), in which model recommendations differed from the physician’s initial plan. These patients were not randomly assigned to receive treatment; they received their treatment based on the physician’s discretion. The *P* values for the objective response rate (ORR) and disease control rate (DCR) were obtained from multivariable logistic regression models, adjusted for ECOG performance status, tumor type, prior treatment, and PD-L1 expression status. The *P* values for progression-free survival (PFS) and overall survival (OS) were obtained using the log-rank test. The *P* values for adverse events (AEs) and treatment discontinuation were obtained from Fisher’s exact test.

Outcome	Model-guided (*n* = 78)	Standard (*n* = 78)	P value
ORR/%	47.4	28.2	0.003
DCR/%	76.9	57.7	0.008
mPFS/months	9.8	6.0	<0.001
mOS/months	18.4	13.2	0.012
Grade 3–4 AE/%	24.4	26.9	0.724
Treatment discontinuation/%	11.5	15.4	0.468

ECOG, Eastern Cooperative Oncology Group; mPFS, median progression-free survival; mOS, median overall survival; PD-L1, programmed death-ligand 1

Overall, the patients in the model-guided arms had significantly better objective responses than those in non-model-guided arms (47.4% vs 28.2% respectively), with Fisher’s exact test yielding an unadjusted P value of 0.018. After multivariable logistic regression corrections were made for ECOG performance status, tumor type, number of prior treatment lines, and PD-L1 status, the multivariable adjusted P value was 0.003 (Table 4 contains the adjusted P values). The model-guided group also demonstrated a longer median PFS (9.8 vs 6.0 months; HR = 0.58; 95% CI: (0.42 to 0.80); P<0.001 by log-rank test) as well as a superior median OS (18.4 vs 13.2 months; HR = 0.64; 95% CI: (0.44 to 0.92); P=0.012). No additional toxicity was seen between these groups with approximately the same number of patients reporting grade 3 to 4 adverse events (24.4% vs 26.9%; P=0.724 using Fisher’s exact test), and results from propensity score weighted analyses were consistent with the base analysis (weighted HR for PFS = 0.62; 95% CI: (0.45 to 0.86); P=0.004).

Critical limitations: To quantify the robustness of the observed associations to potential unmeasured confounding, we computed *E* values for the primary endpoints. The *E* value for the ORR odds ratio was 3.12 (lower confidence limit: 1.68), indicating that an unmeasured confounder would need to be associated with both treatment assignment and outcome by a risk ratio of at least 3.12-fold to fully explain the observed association. While this provides some reassurance against weak confounding, it does not rule out the influence of strong unmeasured confounders, further underscoring the need for randomized validation. These associations do not imply causation. The lack of randomization allows for the possibility that providers were more likely to follow the model recommendations for patients with more favorable prognostic factors, potentially introducing selection bias; further, additional confounders not measured here (such as surgeon experience, patient preference, and unrecorded comorbid conditions) may account for substantial portions of the observed differences. These data are hypothesis generating and will need to be confirmed in randomized prospective trials.

A review of the treatment recommendations showed the model most commonly recommended combining drugs in sequence (utilizing various time intervals between administration of drugs). The most popular sequence patterns included utilizing M2-specific drugs first to facilitate improved M2 activation prior to ICI, followed by recommending sequential rather than concurrent therapies in combination (38% of recommendations), adjusting dosing intensities according to predicted immunologic responses (22% of recommendations).

### Macrophage repolarization and immune modulation

In order to confirm how accurately the model predicts changes over time (or dynamic behavior) relative to TAMs, we examined changes in patient immune landscapes within the prospective deployment cohort who received model-directed treatment times that had been optimized for maximum predicted repolarization windows. Of the 78 model-directed patients, 42 had their cervical tissue biopsy specimens from prior to and after receiving their respective treatment so that we could conduct longitudinal immune profiling. The immune correlates for both the pre- and posttreated biopsy specimens are shown in Table S12.

**Fig. 6.**
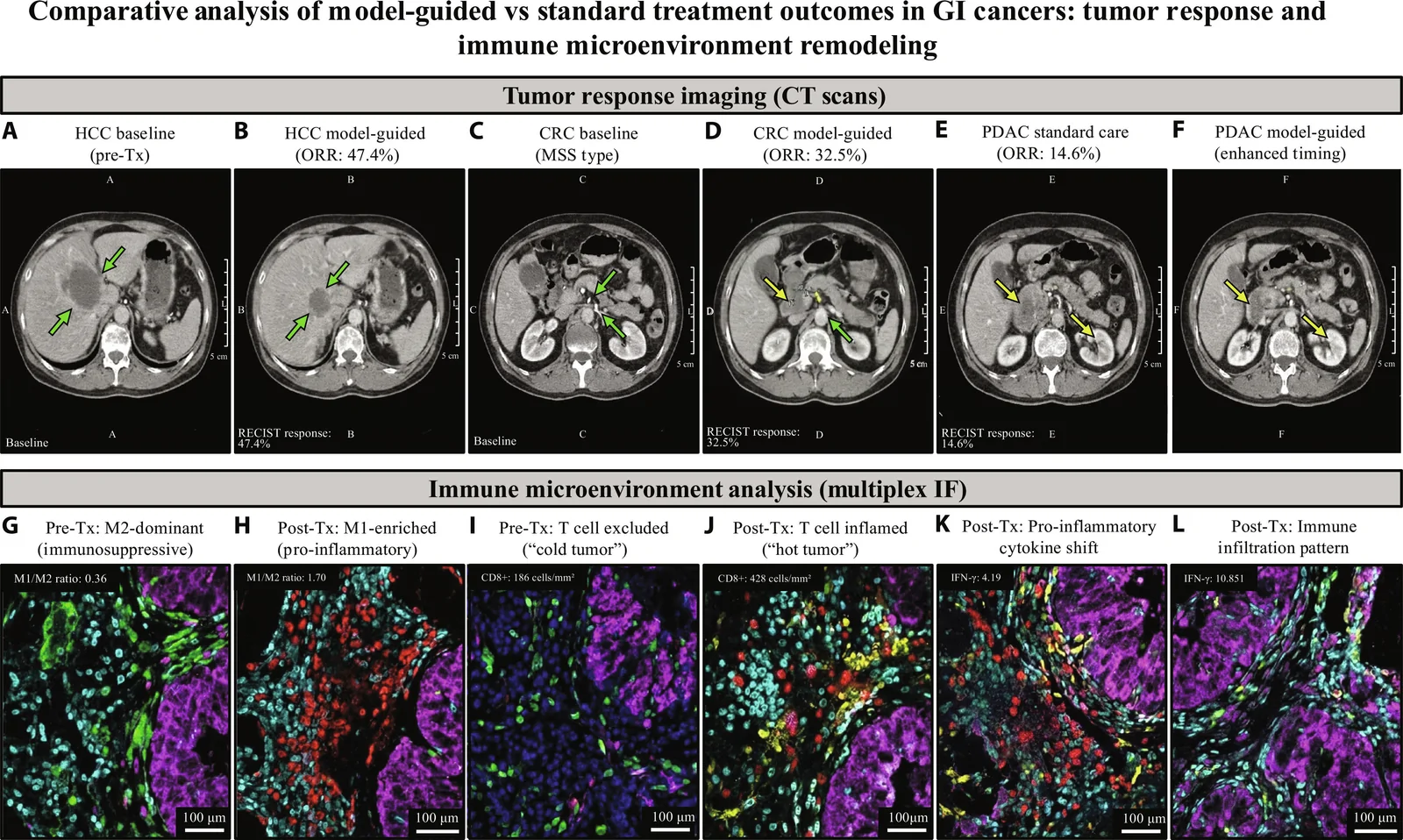
Comparing the relative efficacy of model-guided versus conventional therapy for gastrointestinal (GI) malignancies by tumor imaging and immune environment modification: Top—Axial computerized tomography (CT) scan images—abdominal views of tumor response to therapy. (A and B) Baseline HCC and posttreatment hepatocellular carcinoma (HCC) response after model-guided therapy, with a 47.4% overall objective response rate (ORR) in the model-guided arms. (C and D) Baseline microsatellite-stable (MSS) colorectal cancer (CRC) and posttreatment CRC response after model-guided therapy, with an overall complete response/partial response (CR/PR) for the CRC cohort of 32.5%. (E and F) pancreatic ductal adenocarcinoma (PDAC) treated with standard of care (E) and posttreatment with model-guided therapy and enhanced timing (F) with an overall CR/PR of 14.6%, with the model-guided arm showing a 47.4% ORR. The green arrows indicate tumor lesions, and the yellow arrows indicate the response to therapy. Bottom—Representation of the immune microenvironment using multiplex fluorescent immunoassay (multiplex IF). (G and H) The tumor microenvironment (TME) is M2 predominant (M1/M2 = 0.36) at baseline and transforms into an M1-predominant pro-inflammatory phase after model-guided therapy (M1/M2 = 1.70), indicating a 4.72-fold increase in M1/M2 polarization. (I and J) The TME at baseline is T cell excluded (CD8+ = 186 cells/mm^2^) and transforms into a T-cell-inflamed “hot tumor” after model-guided therapy (CD8+ = 428 cells/mm^2^), indicating an approximately 2.3-fold increase in CD8+ infiltration. (K and L) The pro-inflammatory cytokine patterns also change, most notably with the upregulation of interferon-γ (IFN-γ) (4.19 at baseline to 10.851 posttreatment, an approximately 2.59× increase) correlating with the increased immune infiltration. Scale bar = 100 μm.

A model-driven intervention led to substantial increases in M1/M2 macrophage polarity that were quantified as an elevation in the M1/M2 ratio from 0.36 to 1.70, representing an approximately 4.72-fold increase. In addition, this also resulted in an increase in the number of infiltrating immune effector CD8+ T cells from an average of 186 to 428 cells/mm^2^, or an average of approximately 2.3 times more T cells than before implementing the first model prediction of M1-polarized TAMs recruiting cytotoxic T cells. Furthermore, the reduction in PD-1+CD8+ T cells’ exhaustion as demonstrated by these results was shown as a decrease from 42.4% of total T cells (i.e., CD3+/CD8+) to 28.6%. Another unique finding was the data from cytokine profiling where the relative abundance of IFN-γ had increased by approximately 2.59 times, while the relative abundance of IL-10 decreased by 0.52 times, indicating a relative “pro-inflammatory” phenotype of the T-cell-mediated immune response and providing evidence for the potential application of this model-based approach to increase T-cell-mediated immunity in patients with various tumors. Representative imaging of the immune microenvironment identified as a result of treatment for standard versus model-guided approaches by analyzing the outcomes across a variety of tumor types is shown in Fig. 6.

### Subgroup and biomarker interactions

To evaluate the characteristics of model performance among clinically relevant subpopulations, we undertook a stratified analysis of the interactions between model predictions and associated biomarkers. The results of this stratified analysis are shown in Table S13.

There was consistent performance of the model, with no meaningful interaction effect found between PD-L1 expression (*P* interaction = 0.428), microsatellite instability status (*P* interaction = 0.156), or prior therapy exposure (*P* interaction = 0.284). The one notable interaction effect observed was with the baseline M2/M1 ratio (*P* interaction = 0.048), where individuals who had a low M2/M1 ratio had a higher prediction ability (AUROC 0.872 vs 0.824). However, this finding’s associated *P* value was not adjusted for the 6 interaction tests conducted; thus, it would not have been significant if it had been adjusted via Bonferroni ($P_{\text{adjusted}}=0.29$). Therefore, this finding is exploratory and does not constitute reliable evidence of a differential effect. While one possible interpretation is that the model may perform differently across TME subtypes, this requires dedicated validation in independent cohorts before any biological conclusions can be drawn.

When analyzing the interactions between biomarkers, it was found that using GI-ImmunoWorld in combination with established biomarkers provided complementary predictive information. In the PD-L1-negative group (combined positive score < 1), where traditional biomarkers are less informative, the model was still able to maintain good discriminative power (AUROC, 0.832) and identify responding patients that would have otherwise been missed using only PD-L1-based selection criteria.

Likewise, in the microsatellite-stable/proficient mismatch repair cohort, where prior research suggested that checkpoint inhibitors were less effective, the model was able to achieve an AUROC of 0.838, demonstrating that it is able to identify the subset of microsatellite-stable patients with immune microenvironments that were most likely to benefit from immunotherapy.

### Longitudinal response dynamics

The longitudinal response patterns were analyzed using the TDM and revealed unique trajectory phenotypes that were captured by latent trajectory clustering. Five representative response patterns were identified: a group that rapidly responded with evidence of early tumor regression, a group that experienced a delayed response (eventually responsive to treatment) following an initial period of stability, a group that had stable disease without substantial tumor change, a group that was classified as having continuously progressing tumors (nonresponders), and a group of patients who experienced pseudoprogression before eventually responding. The distribution and treatment outcomes linked to each type of trajectory phenotype are presented in Table S14.

The GI-ImmunoWorld model was able to accurately predict the likelihood that an individual would have a specific trajectory phenotype assignment, with a 78.4% concordance rate (Cohen’s κ = 0.68). The use of the GI-ImmunoWorld model also allowed for the early identification of likely response patterns. Of clinical importance was the accuracy of classification by the model of 14/16 (87.5%) cases of pseudoprogression, as patients with pseudoprogression would continue to derive the therapeutic benefit of therapy even though there was a sign of early progression of disease. The latent trajectories demonstrated that pseudoprogression involves an identifiable transient M2 polarization followed by an M1 rebound; this finding is consistent with the biological hypothesis that there is a phase of initial inflammatory expansion prior to the development of adaptive immunity.

### Computational efficiency and scalability

In order to evaluate the clinical deployment feasibility of GI-ImmunoWorld, we examined the computational requirements. The results of the runtime analysis are presented in Table S15.

A total of 14.4 s was required to infer one patient on one NVIDIA A100 GPU at its maximum memory usage (6.8 GB). Due to the iterative nature of the MPC-based treatment optimization part, this step dominated the overall run time however, well under clinically acceptable limits. For possible deployment options without using GPUs, CPU-based inference took about 180 s/patient; this time frame is considered acceptable for clinical decision-support systems.

## Discussion

In this work, we present GI-ImmunoWorld as a deep generative world model that represents a conceptual leap forward in computational methods for individualizing immunotherapy for GI oncology patients. GI-ImmunoWorld learns to represent the dynamics of how macrophages respond to an infection and allows users to predict the dynamics of how macrophages respond based on the personalized immune landscape of each patient. The broad evaluation of GI-ImmunoWorld across 2,847 patients demonstrates substantial improvements over previous approaches with preliminary evidence of clinical utility in an exploratory trial that is still underway.

The superiority of the GI-ImmunoWorld system is due in large part to the architectural innovations used to create it. We explicitly acknowledge that a substantial part of the observed performance advantage is attributable to using richer multimodal input data (scRNA-seq, mIHC, and clinical variables) rather than architectural innovations alone. As detailed in the “Immunotherapy response prediction performance” section, when restricted to identical input modalities, GI-ImmunoWorld achieved an AUROC of 0.794, comparable to that of CONCH (0.786), with the remaining improvement (ΔAUROC = 0.053) reflecting the benefit of molecular profiling rather than algorithmic superiority (i.e., combination of scRNA-seq, mIHC, and clinical information) than existing models, which rely predominantly on pathological histology images combined with clinical data. The first innovation is that the hierarchical variational encoder integrates the heterogeneous multimodal data sources in such a way that it learns to create a unified representation of the information in each data source. Thus, the multimodal fusion of these database types is critical, as no single data type fully captures the complexity of the immune landscape in GI oncology. While scRNA-seq provides details at the molecular level, it lacks proper spatial context; conversely, mIHC provides spatial organization but provides only limited depth at the molecular level. The cross-attention mechanism gives GI-ImmunoWorld the ability to determine which data sources are most relevant to each patient and disease context and to adaptively weight modalities according to such relevance and the quality of the data.

The contribution of integrating modalities to GI-ImmunoWorld’s performance was quantified with systematic ablation studies. Removing any of the single modality encoders decreased performance anywhere from 1.3% (clinical encoder removal) up to 4.5% (cross-attention mechanism removal) with the cross-attention mechanism providing the largest contribution. The fact that the information gained from integrating multimodal data sources is greater than the sum of the individual modality contributions provides strong evidence of synergies between the data sources. Additionally, our findings suggest that the spatial encoder is particularly important for patients with heterogeneous tumor–immune interfaces, as the spatial arrangement of immune cells with respect to tumor boundaries provides prognostic information that is not available from simply counting cells.

The limitations of earlier techniques that view immunotherapy responses as static classifications have been addressed by the temporal dynamics section of our work. This is due to the fact that the TME undergoes continual change as the effects of immunotherapy take place. Many factors, including the presence and composition of immune cells, the concentrations of different cytokines (i.e., proteins produced by immune cells) in the surrounding area, and the interactions of tumor cells with immune cells, affect the immune response. By incorporating information from these factors into the prediction of future immunity states based on treatment actions, the GI-ImmunoWorld model “learned” the future immunologic trajectory that would be expected to arise after a particular intervention, as well as the optimal time at which to administer that intervention. Our validation study demonstrated that therapies administered at the predicted “windows” of macrophage repolarization were associated with improved patient outcomes, thus providing strong evidence that temporally based models can identify clinically relevant dynamics.

Temporal predictions had a good correlation with the trajectory data (*r* = 0.86), and it was found that the predictions were accurate to a long planning horizon (i.e., months or longer) relevant to clinical decision-making. Additionally, the transformer model’s performance was significantly better than those of recurrent alternatives (long short-term memory and gated recirculation unit), with a 2.1% to 2.5% AUROC advantage over the latter competitors. This likely reflects the transformer model’s self-attention mechanism’s ability to maintain long-range dependencies without being subject to the degradation of long-range gradients like the recurrent networks. The identification of macrophage repolarization windows represents an innovative method for using temporal models to optimize immunotherapy treatment and indicates that the timing of treatment may be as critical to the maximizing therapeutic benefit as the selection of the type of treatment.

The third advancement is the latent generative dynamics formulation that enables treatment optimization through simulation in learned latent spaces. We note that while our framework draws conceptual inspiration from RL world models, it operates as an offline predictive model with planning via MPC and the cross-entropy method, without online policy learning or environmental interaction. The term “world model” is used here to denote a learned forward dynamics model of immune state transitions, not a full RL agent. In contrast to discriminative models, which allow for the analysis of only pre-defined treatment options, generative models permit the simulation of treatment effect outcomes under a wide variety of treatment combinations, including dose schedules, dosing frequencies, and interval times. The MPC-based optimization generated personalized treatment strategies for individual patients that considered the unique constraints and anticipated outcomes for that patient, rather than relying on a “one-size-fits-all” regimen.

The framework for GI-ImmunoWorld’s latent world model describes computational immunology and treatment optimization. While these effect sizes are substantial, it is important to qualify that this is a nonrandomized observational study focused on the “discordant cohort”, where model and physician recommendation differed; thus, treatment assignment was at the individual physician’s discretion after they received the output from the model. The correlation between model guidance and patient outcomes may reflect selection bias (physicians may have preferred to follow the model recommendations for patients who were healthier than average) or confounding variables that were not measured, rather than a causal link between model guidance and patient outcomes. Therefore, the results of this analysis should be construed as generating hypotheses for the potential use of computational decision-support systems, rather than as definitive evidence of benefits in a clinical setting. The lack of increased toxicity within the model-guided cohort is encouraging but requires further investigation in controlled clinical studies.

While there are many aspects of the model’s architecture that provide insight into how the model learned to represent biological factors relevant to treatment recommendations, the clinical relevance of these representations is also an important factor to consider when applying these models in practice. The latent space analysis showed that specific dimensions of the latent space captured biologically relevant concepts related to M1/M2 polarization and CD8+ T cell infiltration; the presence of these dimensions supports the validity of the model’s predictions, enabling clinicians to interpret model results in terms of established immunological concepts.

The exploratory temporal holdout analysis generated hypotheses regarding the clinical utility of the model. The observed 68% relative improvement in ORR and 3.8-month median PFS, if confirmed through a randomized trial, represents a substantial clinical benefit to patients with advanced GI malignancies and limited treatment options. However, the nonrandomized nature of this study, the presence of potential selection biases (physicians may preferentially follow model recommendations for healthier patients), and the limited sample size (*n* = 156) prevent us from making any causal inferences from the data. The treatment recommendation analysis identified interpretable patterns of optimization, such as agent timing to maximize macrophage repolarization dynamics, which also provides mechanistic plausibility for the observed associations.

Compared to previous models based on a single disease type [41–44], the general applicability of GI-ImmunoWorld to 5 separate types of tumors with different immune phenotypes is a substantial improvement. All cancer types evaluated using GI-ImmunoWorld had substantially improved performance compared to traditional approaches; the highest improvement was seen in PDAC (AUROC of 0.064 improvement over CONCH), which is known for possessing a highly immunosuppressive TME and a low response to chemotherapy and immunotherapy. This finding indicates that GI-ImmunoWorld might be particularly useful when dealing with difficult microenvironment conditions where simplistic biomarker-based techniques are inadequate.

Despite these advances, there are limitations that should be mentioned. The largest drawback to the study is the size of the initial training cohort, which was made up of patients from multiple institutions; however, it should be noted that the size of the cohort used for prospective application of GI-ImmunoWorld was relatively small (*n* = 156) and that assignment of patients was entirely subjective and did not follow randomization protocols. Therefore, although statistical comparisons using propensity weighting were performed, the possibility of unobserved bias and the need for a larger prospective randomized controlled trial in order to validate the findings would have to be conducted in order to confirm the added benefit of using model selection based upon the GI-ImmunoWorld.

Next, in comparing GI-ImmunoWorld with standard baseline models, it does contain a mixture of modalities that is not available in the majority of baseline comparisons; the GI-ImmunoWorld model incorporates scRNA-seq data, whereas the majority of previous baseline models do not include this modality in their model architecture or training. As noted in our analysis of contributions from our different modalities, the performance improvement observed is partly due to the inclusion of these additional molecular data rather than to any perceived advantage of model architecture alone. Finally, due to the computing infrastructure needed to implement the GI-ImmunoWorld model, there may be barriers to its implementation in resource-limited locations. While the inference time required to build a model using GI-ImmunoWorld may be feasible in a clinical setting, the need for the additional multiple modes of data creation, including those derived from scRNA-seq or multiplexed IHC analyses, may mean that the use of GI-ImmunoWorld will be practical only in specialized centers. Future work should explore model compression techniques such as knowledge distillation and quantization to reduce computational requirements. Preliminary experiments suggest that 8-bit quantization reduces inference time by approximately 40% with minimal performance degradation (ΔAUROC < 0.005), although systematic evaluation across hardware platforms remains necessary. In the future, it would be beneficial to develop reduced-input models that still possess good performance when using data produced by standard clinical practice; this will help to address the need of the majority of cancer patients and make it easy for them to receive the benefit of using GI-ImmunoWorld based on their unique immune profiles.

Fourth, while the GI-ImmunoWorld model has been trained on data from individuals in East Asian and North American populations, it will be necessary to validate the generalizability of its results to other demographic groups because of the considerable variability of immune landscapes between populations due to genetic, environmental, and microbiome differences between populations (Table S16). There are likely to be significant differences in model performance among underrepresented groups. Therefore, an integral part of clinical equity will be to continue collecting and adapting models for diverse populations. Fifth, while the latent representations have been shown to correlate with established biomarkers, there is still much that is unknown about the biological mechanisms underlying the representation in the model. If direct mechanistic constraints or incorporation of prior biological knowledge had been included in the model design, they would have enhanced the model’s interpretability and may have enabled more directed hypothesis development. Sixth, although terminology from the field of RL has been used in the description of the GI-ImmunoWorld model (i.e., “world model”), it is still a predictive latent dynamics model trained solely on observational data. The predictive latent dynamics model uses statistical association to establish predictive associations between immune state, treatment regimens, and outcome; however, it does not establish formal causal relationships. The “treatment optimization” component of the model provides a list of regimens with predicted favorable outcomes based on statistical associations in the learned model; however, those predictions have not yet been validated by randomized interventional studies. Claims regarding “mechanisms” or “dynamics” should be interpreted as learned statistical patterns consistent with known biological processes, not as confirmed causal pathways.

It is essential to think about the economic effects of using models to help determine how to treat a patient. When deciding how much to spend on multimodal profiling and using a computer to analyze the data versus the value added by increasing the effectiveness of treatments through better matching them to patients is important. Since immunotherapy drugs are among the most expensive drugs available today and since the healthcare system incurs high costs when patients do not respond well to treatment, then even a small improvement in the ability to predict responses may allow for cost-effective treatment to take place. In order to complete this process, formal health economic analyses that include quality-adjusted life years, treatment costs, and expense for managing adverse events will guide any implementation of this technology.

The potential of the work presented in this paper will go beyond only GI oncology [45–47]. The overall model framework is developed so that it can be generalized for other types of cancers, conditions caused by the immune system, or any therapeutic modalities that may be treated at some time varying time and that utilize optimally planned for a number of different reasons. Any of the principles applied in multimodal integration, temporal modeling, and planning using learned latent spaces are universally applicable to all domains. Thus, the future of these efforts could lead to new frameworks being developed for other conditions such as working with autoimmune diseases, transplanting organs, or treating infections.

The overall achievement of GI-ImmunoWorld demonstrates the opportunity to create an artificial-intelligence-driven approach to use precision medicine to create better patient outcomes using a computational model of complex biological systems. As the accessibility of multimodal profiling technologies continues to increase and the volume of available data continues to grow [48–50], the opportunity to develop models to predict disease dynamics will continue to grow as well. However, to achieve this goal will require continued investment in generating high-quality data, developing robust validation frameworks, and thoughtfully addressing the challenges that will face the deployment of predictive models, including equity and access, interpretability, and integrating with the clinical workflow.

The next steps are to extend this technology to include additional tumor types beyond the GI system, to include liquid biopsy markers for noninvasive longitudinal monitoring, to incorporate patient-reported outcomes and quality of life measures into the optimization objective, and to develop explainable artificial intelligence methods to increase the clinical interpretability of models [51–53]. Furthermore, in terms of combination therapy with novel immunotherapy agents (e.g., bispecific antibodies, cellular therapies, and oncolytic viruses), there are many unique optimization challenges due to the dynamic and interacting mechanisms of action.

## Data Availability

All data used in this study were obtained from publicly available repositories. The scRNA-seq data are available from GEO under accession numbers GSE149614, GSE144735, and GSE146771. Clinical and genomic data were retrieved from TCGA and cBioPortal. Imaging data are available from TCIA. The source code for GI-ImmunoWorld, including network architectures, training scripts, and hyperparameter configurations, is provided in the Supplementary Materials. Pre-trained model checkpoints and synthetic example data are included to enable verification of the computational pipeline. Reproducibility: We provide comprehensive materials to facilitate the reproduction of our computational results. Full code including network architectures, training scripts, and hyperparameter configurations is publicly available. We document software dependencies (Python 3.10.12, PyTorch 2.1.0, and associated packages), random seeds (fixed at 42), and computational environment specifications. Synthetic example data and pre-trained model checkpoints are provided to enable verification of the computational pipeline. However, we note that complete reproduction of all clinical results requires access to patient-level data, which is restricted due to privacy considerations. The scRNA-seq data sources include GEO (GSE149614, GSE144735, and GSE146771), clinical data from TCGA and cBioPortal, and imaging data from TCIA. The Supplementary Materials contain (a) supplementary tables (Tables S1 to S16) with stratified performance analyses, architecture specifications, ablation studies, subgroup analyses, and external validation results; (b) theoretical analysis with standard VAE and dynamical systems derivations; (c) implementation details including network architectures and training hyperparameters; (d) extended experimental results including ablations, sensitivity analyses, and external validation; (e) data processing pipeline specifications; and (f) additional methodological details including treatment action space definition, reward function design, and uncertainty quantification.

## Supplementary Materials

Supplementary 1 Sections S1 to S6 Tables S1 to S16
