# Supplementary material for: Deep Generative Model of Macrophage Immune Response for Hepato-intestinal Tumor Therapy Optimization
Source: Cyborg Bionic Syst. 2026 Jun 16;7:0559. doi: 10.34133/cbsystems.0559 (PMC13505573; doi:10.34133/cbsystems.0559)
Supplement: Supplementary file 1 [file cbsystems.0559.f1.pdf]

Supplementary Materials for:
Deep Generative Model of Macrophage Immune Response for Hepato-Intestinal Tumor Therapy Optimization

Contents

S1 Supplementary Tables **3**

S2 Theoretical Analysis **8**
S2.1 Evidence Lower Bound Derivation . . . 8
S2.2 Temporal Dynamics Consistency . . . 9
S2.3 Relationship to Mutual Information . . . 9
S2.4 Convergence Analysis . . . 10

S3 Implementation Details **10**
S3.1 Network Architectures . . . 10
S3.2 Training Configuration . . . 10
S3.3 Computational Resources . . . 10
S3.4 Software Dependencies . . . 10

S4 Extended Experimental Results **11**
S4.1 Additional Ablation Studies . . . 11
S4.2 Sensitivity Analysis . . . 11
S4.3 Cross-validation Results . . . 11
S4.4 External Validation Cohort . . . 11

S5 Data Processing Pipeline **11**
S5.1 Single-cell RNA Sequencing Processing . . . 11
S5.2 Multiplex Immunohistochemistry Processing . . . 12
S5.3 Clinical Data Harmonization . . . 12
S5.4 Data Augmentation Strategies . . . 12

S6 Additional Methodological Details **13**
S6.1 Treatment Action Space Definition . . . 13
S6.2 Reward Function Design . . . 13

S6.3 Uncertainty Quantification 13

S1 Supplementary Tables

Table S1: Response prediction AUROC stratified by available input modality combinations. Results are reported on the held-out test set.

Available Modalities	N	AUROC	95% CI
scRNA-seq + mIHC + Clinical (all three)	178	0.862	0.824–0.900
mIHC + Clinical (no scRNA-seq)	156	0.794	0.748–0.840
scRNA-seq + Clinical (no mIHC)	52	0.818	0.762–0.874
Clinical only	41	0.724	0.658–0.790
**All test patients (weighted)**	**427**	**0.847**	**0.812–0.878**

Table S2: Network architecture specifications.

Component	Specification
Single-cell Encoder (GAT)	
Input dimension	2,000 (HVGs)
Hidden dimensions	[512, 256, 128]
Number of attention heads	8
Dropout rate	0.2
Aggregation	Mean pooling
Spatial Encoder	
Conv2D layers	[64, 128, 256]
Kernel size	3×3
Transformer layers	4
Attention heads	8
Clinical Encoder	
Input dimension	48
Hidden dimensions	[256, 128]
Activation	GELU
Cross-modal Attention	
Hidden dimension	256
Attention heads	8
Layers	2
Latent Space	
Dimension	64
Prior	Standard Gaussian
Temporal Dynamics (Transformer)	
Layers	6
Hidden dimension	512
Attention heads	8
FFN dimension	2048
Dropout rate	0.1

Table S3: Training hyperparameters.

**Hyperparameter**	**Value**
Optimizer	AdamW
Learning rate	1×10^{-4}
Weight decay	0.01
Batch size	64
Gradient clipping	1.0
β (KL weight)	0.1
λ_{dyn}	0.5
λ_{pred}	1.0
Warmup epochs	10
Total epochs	200
Early stopping patience	20
Learning rate scheduler	Cosine annealing
Mixed precision	FP16

Table S4: Sensitivity analysis of key hyperparameters.

**Parameter**	**Range**	**AUROC**	**Std**
Learning rate	[1e-5, 1e-3]	0.842	0.008
Batch size	[32, 128]	0.844	0.006
β (KL weight)	[0.01, 1.0]	0.838	0.012
Dropout rate	[0.1, 0.3]	0.845	0.004
Attention heads	[4, 16]	0.846	0.003

Table S5: Single-cell RNA sequencing-derived cellular composition of the tumor microenvironment across tumor types.

**Cell Type (% of total)**	**HCC**	**CCA**	**CRC**	**GC**	**PDAC**
Tumor cells	42.3	48.6	38.4	44.2	52.1
TAMs (total)	18.6	15.2	16.8	14.4	12.3
M1-like	4.8	3.2	5.6	4.1	2.4
M2-like	8.2	7.8	6.4	6.2	6.8
Transitional	5.6	4.2	4.8	4.1	3.1
CD8+ T cells	12.4	8.6	14.2	11.8	6.4
CD4+ T cells	8.2	6.8	9.4	8.2	5.8
Tregs	3.4	4.2	2.8	3.6	4.8
B cells	4.2	3.8	5.6	4.4	3.2
Fibroblasts	6.8	8.4	7.2	8.2	10.4
Endothelial	4.1	4.4	5.6	5.2	5.0

Table S6: Extended ablation study results on response prediction.

**Model Variant**	**AUROC**	Δ
**GI-ImmunoWorld (Full)**	**0.847**	–
Encoder Ablations		
w/o GAT (use MLP)	0.812	-0.035
w/o Spatial encoder	0.818	-0.029
w/o Clinical encoder	0.834	-0.013
w/o Cross-attention	0.802	-0.045
Concatenation fusion	0.808	-0.039
Latent Space Ablations		
Latent dim = 32	0.828	-0.019
Latent dim = 128	0.842	-0.005
Standard VAE (β=1)	0.824	-0.023
$\beta = 0.5$	0.838	-0.009
Temporal Module Ablations		
w/o Temporal (static)	0.812	-0.035
LSTM instead of Transformer	0.826	-0.021
GRU instead of Transformer	0.822	-0.025
2 Transformer layers	0.832	-0.015
Training Ablations		
w/o Multi-task learning	0.828	-0.019
w/o Data augmentation	0.836	-0.011

Table S7: Five-fold cross-validation results for response prediction.

**Fold**	**AUROC**	**AUPRC**	**Acc**	**F1**
1	0.852	0.632	0.788	0.718
2	0.838	0.614	0.774	0.704
3	0.856	0.642	0.792	0.726
4	0.842	0.618	0.778	0.708
5	0.848	0.626	0.784	0.714
**Mean**	**0.847**	**0.626**	**0.783**	**0.714**
**Std**	0.007	0.011	0.007	0.009

Table S8: Response prediction performance when all methods use identical input modalities (mIHC-derived features + clinical variables only, no scRNA-seq). This comparison isolates architectural contributions from modality advantages. Evaluated on the subset of test patients with mIHC and clinical data available (n = 323).

**Method**	**AUROC**	**AUPRC**	**Sensitivity**	**F1**
MCAT	0.756	0.512	0.642	0.594
PORPOISE	0.768	0.528	0.658	0.612
TransMIL	0.742	0.494	0.628	0.578
SurvPath	0.774	0.536	0.664	0.618
CONCH	0.786	0.554	0.678	0.634
**GI-ImmunoWorld (mIHC + Clinical)**	**0.794**	**0.568**	**0.688**	**0.648**

Table S9: Correlation between latent dimensions and immune markers. Pearson correlation coefficients shown.

**Latent Dim**	**M1 Score**	**M2 Score**	**CD8 Inf**	**TIS**	**PD-L1**
z_1	0.72	-0.68	0.64	0.58	0.42
z_2	-0.64	0.76	-0.52	-0.48	0.28
z_3	0.48	-0.32	0.82	0.68	0.36
z_4	0.36	0.42	0.28	0.24	0.74
z_5	0.28	-0.24	0.36	0.78	0.32

Table S10: Response prediction AUROC stratified by tumor type.

**Method**	**HCC**	**CCA**	**CRC**	**GC**	**PDAC**
Combined biomarkers	0.708	0.654	0.724	0.682	0.628
TIDE	0.686	0.642	0.708	0.664	0.612
MCAT	0.768	0.724	0.782	0.748	0.702
PORPOISE	0.782	0.738	0.794	0.762	0.718
CONCH	0.798	0.756	0.812	0.778	0.734
**GI-ImmunoWorld**	**0.862**	**0.818**	**0.874**	**0.842**	**0.798**

Table S11: Temporal trajectory prediction accuracy for immune dynamics. MSE: mean squared error, MAE: mean absolute error.

**Predicted Variable**	**MSE**	**MAE**	**Corr**	R^2
M1/M2 ratio	0.042	0.156	0.84	0.71
CD8+ T cell density	0.038	0.142	0.86	0.74
Treg proportion	0.028	0.124	0.82	0.68
IFN-γ signature	0.034	0.138	0.88	0.76
Exhaustion score	0.046	0.168	0.78	0.62
**Overall (latent z)**	0.036	0.144	0.86	0.72

Table S12: Immune landscape changes following model-guided macrophage repolarization interventions in the prospective deployment cohort ($n = 42$ with paired biopsies).

**Immune Marker**	**Pre-Tx**	**Post-Tx**	**Fold Change**
M1 TAMs (cells/mm^2)	124 $\pm$ 38	286 $\pm$ 72	2.31
M2 TAMs (cells/mm^2)	342 $\pm$ 86	168 $\pm$ 54	0.49
M1/M2 ratio	0.36 $\pm$ 0.12	1.70 $\pm$ 0.48	4.72
CD8+ T cells (cells/mm^2)	186 $\pm$ 62	428 $\pm$ 98	2.30
PD-1+ CD8+ (%)	42.4 $\pm$ 12.8	28.6 $\pm$ 8.4	0.67
IFN-γ (pg/mL)	18.6 $\pm$ 6.4	48.2 $\pm$ 14.2	2.59
IL-10 (pg/mL)	42.8 $\pm$ 12.6	22.4 $\pm$ 8.2	0.52

Table S13: Response prediction AUROC stratified by clinical and molecular subgroups.

Subgroup	N	AUROC	95% CI	p-int
PD-L1 Expression				
CPS < 1	186	0.832	0.774-0.890	0.428
CPS ≥ 1	241	0.858	0.812-0.904	
MSI Status				
MSS/pMMR	389	0.838	0.798-0.878	0.156
MSI-H/dMMR	38	0.892	0.824-0.960	
TMB Level				
TMB < 10	312	0.842	0.798-0.886	0.318
TMB ≥ 10	115	0.864	0.808-0.920	
Prior Systemic Therapy				
Treatment-naive	198	0.856	0.806-0.906	0.284
Prior therapy	229	0.838	0.788-0.888	
ECOG Performance Status				
PS 0	142	0.868	0.814-0.922	0.092
PS 1-2	285	0.834	0.790-0.878	
Baseline M2/M1 Ratio				
Low ($<$ median)	214	0.872	0.826-0.918	0.048
High ($\geq$ median)	213	0.824	0.772-0.876	

Table S14: Response trajectory phenotypes and associated outcomes.

Trajectory Phenotype	N (%)	BOR (CR/PR)	mPFS (mo)	mOS (mo)
Rapid responder	89 (20.8)	68.5%	14.2	26.8
Delayed responder	62 (14.5)	54.8%	11.6	22.4
Stable disease	124 (29.0)	12.9%	7.8	16.2
Non-responder	136 (31.9)	2.2%	3.4	9.6
Pseudoprogression	16 (3.8)	62.5%	12.8	24.6
**Total**	427	24.6%	6.4	13.8

Table S15: Computational efficiency analysis.

Operation	Time (s)	Memory (GB)
Single-cell encoding (10K cells)	2.4	4.2
Spatial feature extraction	1.8	2.8
Multi-modal fusion	0.6	1.2
Temporal rollout (12 steps)	1.2	0.8
Treatment optimization (100 iter)	8.4	1.6
**Total inference**	14.4	6.8

Table S16: External validation cohort results ($n = 186$).

**Metric**	**Internal Test**	**External**	Δ
AUROC	0.847	0.824	-0.023
AUPRC	0.624	0.598	-0.026
Accuracy	0.782	0.758	-0.024
Sensitivity	0.738	0.712	-0.026
Specificity	0.806	0.784	-0.022
F1 Score	0.712	0.686	-0.026
C-index (survival)	0.786	0.762	-0.024

S2 Theoretical Analysis

This appendix presents the mathematical foundations underlying GI-ImmunoWorld. The derivations follow standard variational inference and dynamical systems theory; we do not claim theoretical novelty but include them for completeness and to establish notation connecting our implementation to the broader VAE and world model literature. Readers familiar with these foundations may proceed directly to the implementation details in Section S3.

S2.1 Evidence Lower Bound Derivation

We derive the evidence lower bound (ELBO) for the GI-ImmunoWorld generative model. Let $\mathbf{X} = \{\mathbf{X}^{(s)}, \mathbf{X}^{(m)}, \mathbf{X}^{(c)}\}$ denote the observed multi-modal data and $\mathbf{z}$ denote the latent representation. The marginal likelihood is:

$$\log p(\mathbf{X}) = \log \int p(\mathbf{X}|\mathbf{z})p(\mathbf{z})d\mathbf{z} \tag{S1}$$

Introducing the variational posterior $q_\phi(\mathbf{z}|\mathbf{X})$:

$$\begin{aligned}\log p(\mathbf{X}) = \mathbb{E}_{q_\phi}\left[\log \frac{p(\mathbf{X},\mathbf{z})}{q_\phi(\mathbf{z}|\mathbf{X})}\right] \\ + D_{\mathrm{KL}}(q_\phi(\mathbf{z}|\mathbf{X}) \| p(\mathbf{z}|\mathbf{X}))\end{aligned} \tag{S2}$$

Since the KL divergence is non-negative, we obtain:

$$\begin{aligned}\log p(\mathbf{X}) \geq \mathbb{E}_{q_\phi}\left[\log p(\mathbf{X}|\mathbf{z})\right] \\ - D_{\mathrm{KL}}(q_\phi(\mathbf{z}|\mathbf{X}) \| p(\mathbf{z})) = \mathcal{L}(\phi, \psi; \mathbf{X})\end{aligned} \tag{S3}$$

For the hierarchical structure with modality-specific latents $\{\mathbf{z}^{(s)}, \mathbf{z}^{(m)}, \mathbf{z}^{(c)}\}$ and shared latent

$\mathbf{z}$, the ELBO factorizes as:

$$\begin{aligned}\mathcal{L} = \sum_{k\in\{s,m,c\}} & \mathbb{E}_{q_\phi}\left[\log p(\mathbf{X}^{(k)}|\mathbf{z}^{(k)})\right] \\ & - \beta \cdot D_{\mathrm{KL}}(q_\phi(\mathbf{z}|\mathbf{X})\|p(\mathbf{z})) \\ & - \sum_k D_{\mathrm{KL}}(q_\phi(\mathbf{z}^{(k)}|\mathbf{X}^{(k)})\|p(\mathbf{z}^{(k)}|\mathbf{z}))\end{aligned} \tag{S4}$$

The β coefficient controls the trade-off between reconstruction fidelity and latent space regularity. In the classical β-VAE formulation, $\beta > 1$ encourages disentanglement by strengthening the KL penalty. In our setting, after hyperparameter optimization, we found $\beta = 0.1$ yielded the best predictive performance, effectively prioritizing reconstruction and prediction accuracy while still maintaining meaningful latent structure. This choice reflects our primary objective of clinical prediction rather than unsupervised disentanglement, and we therefore describe our approach as a "KL-weighted VAE" rather than a strict β-VAE.

S2.2 Temporal Dynamics Consistency

Proposition S1. *For a Markovian transition model $p_\theta(\mathbf{z}_{t+1}|\mathbf{z}_t, a_t)$ with bounded Lipschitz constant L, the multi-step prediction error is bounded by:*

$$\|\hat{\mathbf{z}}_T - \mathbf{z}_T\|_2 \leq \frac{L^T - 1}{L - 1} \cdot \epsilon \tag{S5}$$

where ϵ bounds the single-step prediction error.

Proof. Let $\epsilon_t = \|\hat{\mathbf{z}}_t - \mathbf{z}_t\|_2$ denote the prediction error at step t. By the Lipschitz condition:

$$\begin{aligned}\epsilon_{t+1} &= \|f_\theta(\hat{\mathbf{z}}_t, a_t) - f_\theta(\mathbf{z}_t, a_t) \\ &\quad + f_\theta(\mathbf{z}_t, a_t) - \mathbf{z}_{t+1}\|_2 \\ &\leq L \cdot \epsilon_t + \epsilon\end{aligned} \tag{S6}$$

Solving this recurrence with $\epsilon_0 = 0$:

$$\epsilon_T \leq \epsilon \sum_{t=0}^{T-1} L^t = \frac{L^T - 1}{L - 1} \cdot \epsilon \tag{S7}$$

This bound motivates regularization strategies to control the Lipschitz constant of the transition model and minimize single-step prediction error.

S2.3 Relationship to Mutual Information

Following standard VAE analysis, maximizing the ELBO implicitly constrains the mutual information between data and latent representations. Specifically, the KL term in the ELBO can be

decomposed as:

$$\mathbb{E}_{p(\mathbf{X})}[D_{\mathrm{KL}}(q_\phi(\mathbf{z}|\mathbf{X})\|p(\mathbf{z}))] = I_q(\mathbf{X};\mathbf{z}) + D_{\mathrm{KL}}(q_\phi(\mathbf{z})\|p(\mathbf{z})) \tag{S8}$$

where $I_q(\mathbf{X};\mathbf{z})$ is the mutual information under the variational distribution $q_\phi(\mathbf{z}) = \mathbb{E}_{p(\mathbf{X})}[q_\phi(\mathbf{z}|\mathbf{X})]$, this means that training with a KL-weighted objective gives rise to informative but regularized representations; in particular, we find that with $\beta = 0.1$ a KL penalty that is relatively weak allows the model to capture much more information from its input (which ultimately aids predictive performance), but at the expense of potentially less regularized latent geometry.

S2.4 Convergence Analysis

Lemma S1. *Under standard regularity conditions (smooth loss landscape, bounded gradients), the composite training objective converges to a stationary point at rate $O(1/\sqrt{T})$ where T is the number of optimization steps.*

The proof follows from standard convergence results for stochastic gradient descent with Adam optimizer, noting that the composite objective decomposes into smooth component losses.

S3 Implementation Details

S3.1 Network Architectures

The complete architectural specifications for each model component are provided in Table S2.

S3.2 Training Configuration

The training hyperparameters are summarized in Table S3.

S3.3 Computational Resources

Model training was conducted on a cluster of 8 NVIDIA A100 (80GB) GPUs with data parallel distributed training. Total training time was approximately 72 hours. Inference was performed on a single A100 GPU with average latency of 14.4 seconds per patient.

S3.4 Software Dependencies

The implementation used the following software versions: Python 3.10.12, PyTorch 2.1.0, PyTorch Geometric 2.4.0, Transformers 4.35.2, scanpy 1.9.6, scikit-learn 1.3.2, numpy 1.24.3, pandas 2.1.1, and CUDA 12.1. Random seeds were fixed at 42 for all experiments to ensure reproducibility.

S4 Extended Experimental Results

S4.1 Additional Ablation Studies

We conducted comprehensive ablation studies to assess the contribution of individual model components. Table S6 presents extended results.

S4.2 Sensitivity Analysis

We evaluated model robustness to hyperparameter variations. Table S4 presents the sensitivity analysis results.

The model demonstrated robust performance across reasonable hyperparameter ranges, with standard deviations below 0.015 for all parameters tested.

S4.3 Cross-validation Results

Five-fold cross-validation was performed to assess model stability. Table S7 presents the results.

The low standard deviations (AUROC std = 0.007) indicate stable performance across different data splits.

S4.4 External Validation Cohort

To assess whether GI-ImmunoWorld is valid across other institutions, we tested the model using a sample of patients from three different sites that did not use GI-ImmunoWorld during the development of the model (n = 186). Most of the patients in the external validation sample (78 patients from Germany, 62 patients from France, and 46 patients from Australia) had similarly distributed tumors to those in the original GI-ImmunoWorld dataset (24% HCC, 15% CCA, 32% CRC, 18% GC, and 11% PDAC), and all patients had mIHC data available, along with clinical data but only 89 patients (47.8%) had scRNA-seq data available for analysis. Despite some demographic differentials (the median age of this cohort was older than the training sample; 66 years vs. 62 years), and a higher percentage of the cohort having received prior chemotherapy (58% of this cohort vs. 45% of the training sample), the results of the external validation cohort were comparable to those in the original development cohort (Table S16).

The model maintained strong performance on the external cohort with modest degradation (AUROC 0.824 vs. 0.847, Δ = -0.023), demonstrating generalizability across independent patient populations and institutional practices.

S5 Data Processing Pipeline

S5.1 Single-cell RNA Sequencing Processing

The following pipeline was utilized to process raw single-cell RNA sequencing data (scRNA-seq). Cell Ranger (Version 7.0) was used to align Reads against the GRCh38 (Genome Reference Consortium

Human Build 38) reference genome. Quality Control filters were applied (minimum of 500 genes per cell); maximum of 20% Mitochondrial DNA content and detection of Doublets using Scrublet with a expected doublet rate of 0.06. Normalization accounted for the size factors calculated from pooled scran counts after which log transformation was performed. Highly Variable Genes (HVGs) were identified using the Seurat (Version 4.0) method, with a total of 2000 genes being identified as HVGs. Due to Batch effects, using the Harmony method (with study site being used as the batch variable), batch correction was performed on the raw gene expression matrix after normalization. To visualize the high-dimensional data resulting from scRNA-seq, PCA (Principal Components 50) was run followed by UMAP (Uniform Manifold Approximation and Projection). Cell types were annotated based upon known marker genes; a hierarchical assignment with validation from the expert pathologist was completed.

S5.2 Multiplex Immunohistochemistry Processing

HALO v3.4 software was used to process and analyze the mIHC slides with HALO's artificial intelligence (AI) nuclear segmentation algorithm on the cells. In this study, the phenotype of the cells were determined by manual validation of the images after thresholding on 10% of all images and then determining by criteria which images showed the presence or absence of a cell type. Cellular density (cells/mm2), distances from the nearest neighbor and tests for spatial interaction were calculated using spatstat package in R.

S5.3 Clinical Data Harmonization

Data for clinical variables from multiple sites were standardized and harmonized by developing common definitions of the clinical variables across multiple sites. Missing clinical variables (8.3% of each variable) from each clinical site were filled in using MICE (Multiple Imputation using Chaining). Continuous clinical variables were adjusted to a mean of 0 and a variance of 1, and categorical clinical variables were created by the one-hot encode process.

S5.4 Data Augmentation Strategies

We used many different methods to augment the amount of data available for training our models, in order to help make them more robust to variations in the training data and reduce overfitting. For scRNA-seq, we implemented dropout augmentation (masking 10% of the gene expression values randomly), Gaussian noise ($\sigma = 0.1$), and randomly sampled subsets of cells (80-100% of the number of cells in each sample). For the spatial features, we used random rotations (0-360°), horizontal and vertical flips, and random cropping with a minimum of 90% area retained. We did not augment any of the clinical variables because we wanted to maintain interpretability.

S6 Additional Methodological Details

S6.1 Treatment Action Space Definition

The total number of combinations for the treatment action space $\mathcal{A}$ is 847 combinations that have been clinically validated as appropriate treatment regimens. As stated above, the action space was created based on principles stated in the current international clinical guidelines (NCCN, ESMO, CSCO) and clinical practice. The treatment action space consists of anti-PD-1 monotherapy (pembrolizumab, nivolumab, camrelizumab), anti-PD-L1 monotherapy (atezolizumab, durvalumab), anti-CTLA-4 agents (ipilimumab, tremelimumab), tyrosine kinase inhibitors (lenvatinib, sorafenib, regorafenib, cabozantinib), VEGF inhibitors (bevacizumab, ramucirumab), cytotoxic chemotherapy backbones (FOLFOX, FOLFIRI, gemcitabine-cisplatin), dosing levels (standard and reduced per label recommendations), and timing modifiers (concurrent and sequential) as specified in treatment protocols. All recommendations were reviewed by the multidisciplinary tumor board prior to being implemented; the board's recommendation must not be overridden by physician judgment based on the individual patient's clinical circumstances.

S6.2 Reward Function Design

The components of the reward function are defined as follows; the reward associated with response $R_{\text{response}}(\mathbf{z}_t)$ is calculated by using the softmax probability of the response category from the output layer of the model, the macrophage polarization reward $R_{\text{TAM}}(\mathbf{z}_t)$ represents a measure of favourable macrophage polarisation based on the difference between M1 and M2 latent activations, and is normalised to the interval [-1, 1], the toxicity penalty $R_{\text{toxicity}}(a_t)$ is defined as the expected rates of Gr3/4 adverse events for each treatment as obtained from clinical trials, and finally, the survival reward $R_{\text{survival}}(\mathbf{z}_t)$ is defined as the logarithm of the predicted survival probability at 12 months from the model head that predicts survival.

The values assigned to the weights in the reward function were determined through a grid search on the validation dataset with $w_1 = 1.0$, $w_2 = 0.5$, $w_3 = -0.3$ and $w_4 = 0.8$. The discount factor (γ) was set to 0.95 to provide an appropriate balance between the immediate and longer-term aspect of survival.

S6.3 Uncertainty Quantification

Monte Carlo dropout was used to quantify prediction uncertainty using 50 forward passes during inference. The standard deviation of dropout samples was used to quantify epistemic uncertainty. Patients with the highest uncertainty (top 20% of the uncertainty distribution) were highlighted for additional clinical review, since high uncertainty could reflect atypical immune responses or quality concerns regarding data upon which this judgement is based.
